# Anticancer derivative of the natural alkaloid, theobromine, inhibiting EGFR protein: Computer-aided drug discovery approach

**DOI:** 10.1371/journal.pone.0282586

**Published:** 2023-03-09

**Authors:** Ibrahim H. Eissa, Reda G. Yousef, Eslam B. Elkaeed, Aisha A. Alsfouk, Dalal Z. Husein, Ibrahim M. Ibrahim, Mohamed S. Alesawy, Hazem Elkady, Ahmed M. Metwaly

**Affiliations:** 1 Faculty of Pharmacy (Boys), Pharmaceutical Medicinal Chemistry & Drug Design Department, Al-Azhar University, Cairo, Egypt; 2 Department of Pharmaceutical Sciences, College of Pharmacy, AlMaarefa University, Riyadh, Saudi Arabia; 3 Department of Pharmaceutical Sciences, College of Pharmacy, Princess Nourah Bint Abdulrahman University, Riyadh, Saudi Arabia; 4 Faculty of Science, Chemistry Department, New Valley University, El-Kharja, Egypt; 5 Faculty of Science, Biophysics Department, Cairo University. Cairo, Egypt; 6 Faculty of Pharmacy (Boys), Pharmacognosy and Medicinal Plants Department, Al-Azhar University, Cairo, Egypt; 7 Biopharmaceutical Products Research Department, Genetic Engineering and Biotechnology Research Institute, City of Scientific Research and Technological Applications (SRTA-City), Alexandria, Egypt; Ahram Canadian University, EGYPT

## Abstract

A new semisynthetic derivative of the natural alkaloid, theobromine, has been designed as a lead antiangiogenic compound targeting the EGFR protein. The designed compound is an (*m*-tolyl)acetamide theobromine derivative, (**T-1-MTA**). Molecular Docking studies have shown a great potential for **T-1-MTA** to bind to EGFR. MD studies (100 ns) verified the proposed binding. By MM-GBSA analysis, the exact binding with optimal energy of **T-1-MTA** was also identified. Then, DFT calculations were performed to identify the stability, reactivity, electrostatic potential, and total electron density of **T-1-MTA**. Furthermore, ADMET analysis indicated the **T-1-MTA**’s general likeness and safety. Accordingly, **T-1-MTA** has been synthesized to be examined *in vitro*. Intriguingly, **T-1-MTA** inhibited the EGFR protein with an IC_50_ value of 22.89 nM and demonstrated cytotoxic activities against the two cancer cell lines, A549, and HCT-116, with IC_50_ values of 22.49, and 24.97 μM, respectively. Interestingly, **T-1-MTA**’s IC_50_ against the normal cell lines, WI-38, was very high (55.14 μM) indicating high selectivity degrees of 2.4 and 2.2, respectively. Furthermore, the flow cytometry analysis of A549 treated with **T-1-MTA** showed significantly increased ratios of early apoptosis (from 0.07% to 21.24%) as well as late apoptosis (from 0.73% to 37.97%).

## Introduction

The world health organization (WHO) anticipated that during the next few years, cancer will dominate all other causes of death [1]. Developing treatments that suppress the growth of cancer by interacting with specific molecular targets and damaging the cancer cells is a major concern for medicinal chemists that work on cancer therapy [2]. Increasing vascularity (angiogenesis) is a crucial process that increases tumor development, so anti-angiogenesis strategies were considered to be very effective in the treatment [3]. Also, it was confirmed that the angiogenesis and growth of cancer cells are driven by the epidermal growth factor receptors (EGFR) [4]. In response to EGFR overexpression, downstream signaling pathways stimulate cell proliferation, differentiation, and survival. In cancers, EGFR was found to be elevated and promoted several solid malignant tumors [5]. Numerous cancer types express lower survival rates when EGFR is expressed. Also, EGFR’s expression served as a powerful diagnostic and prognostic indicator for cancer [6]. In contrast, this overexpression allowed researchers to utilize the EGFR’s inhibition as an essential strategy in cancer treatment [7,8].

Anciently, natural products, especially plants, were the most vital bases of treatments [9,10]. Recently, one-third of the FDA-approved drugs from 1981–2014 have been derived from natural sources [11]. Anticancer drug discovery finds xanthines, and xanthine derivatives, to be interesting compounds that exhibit different antimutagenic properties against ovarian cancer [12], prostate cancer [13], breast cancer, and leukemia [14].

Theobromine, the famous natural alkaloid, was discovered in 1841, while the synthesis of theobromine was described in 1882 [15,16]. Theobromine is found primarily in *Theobroma cacao*, chocolate, and other foods including tea leaves [17]. Theobromine showed promising anti-cancer activity *in vitro* and *in vivo* through the inhibition of DNA synthesis in glioblastoma multiforme [18] and prevented lung cancer angiogenesis [19]. Interestingly, in ovarian cancer, theobromine inhibited the VEGF *in vivo* and *in vitro* [20]. By using semi-synthesis to produce analogs, we can discover more potent drugs, give repurposing opportunities, and develop novel bioactive compounds, enhance drug-likeness, and improve pharmacokinetics and pharmacodynamics [21].

In scientific society today, computer-aided drug discovery (CADD) is widely accepted as a means of applying theoretical ideas using computers and a set of techniques for investigating chemical problems and is used in the pharmaceutical industry to investigate how potential drugs interact with biomolecules [22–26]. Our team applied the CADD in molecular design and docking, computational toxicity and ADME [27,28], in addition to MD simulations [29,30].

### Rationale

**Fig 1:**
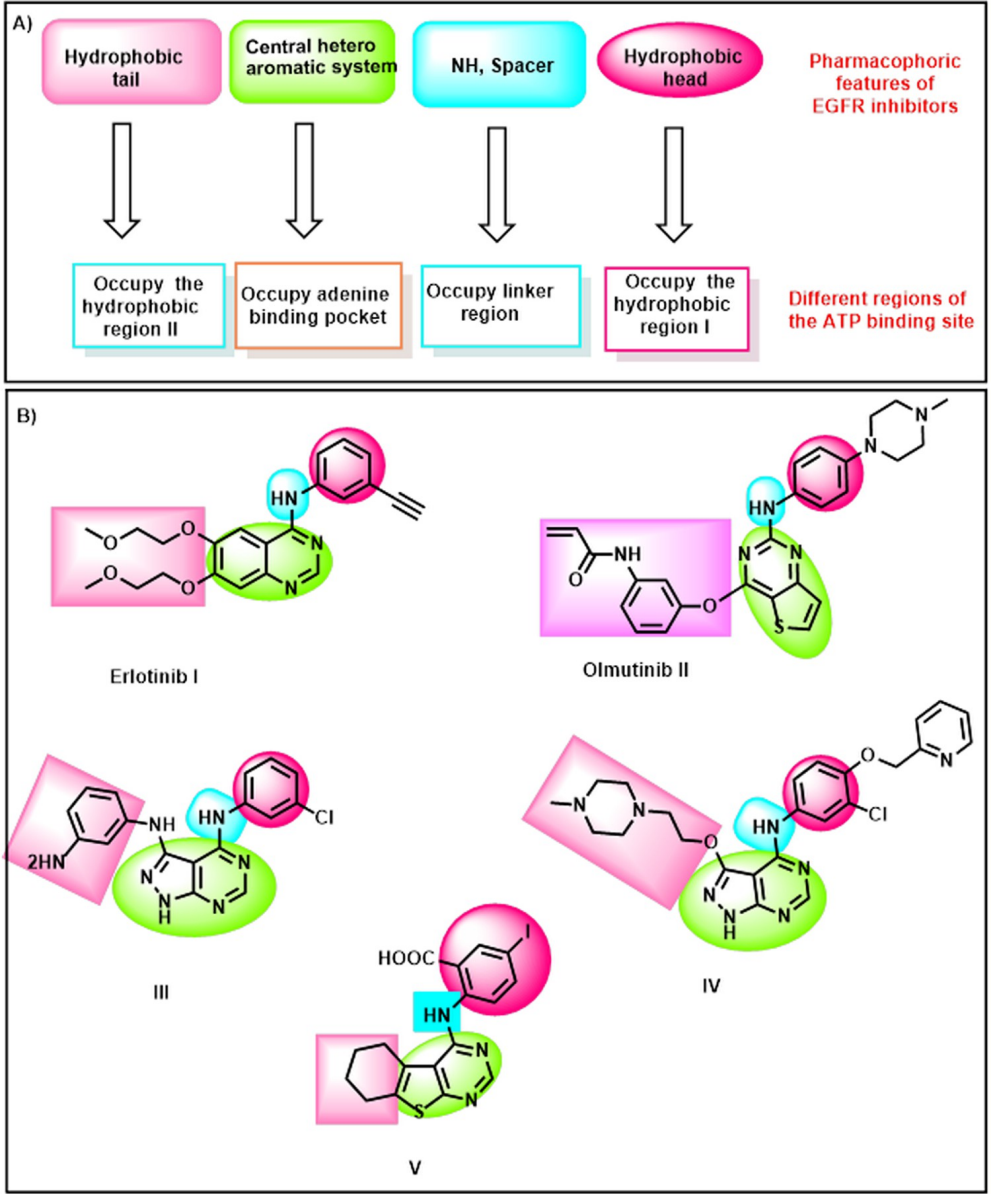
EGFR inhibitors’ pharmacophoric features.

Erlotinib **I** [31,32] and olmutinib **II** [33] are reported **as** EGFR inhibitors. Compounds **III** and **IV** are derivatives of 1*H*-pyrazolo[3,4-*d*]pyrimidine that showed excellent efficacy for inhibiting EGFR-TK at nono-molar doses [34,35]. Our team previously synthesized compound **V** (a thieno[2,3-*d*]pyrimidine derivative) that was promising anti-proliferative and EGFR inhibitor [36] (**Fig 1**).

These compounds possess some pharmacophoric features of EGFR-TKIs. These features are a planar heterocyclic system, an NH spacer, a terminal hydrophobic head and a hydrophobic tail. The key roles of the above-mentioned structural moieties are to occupy the adenine binding pocket [37], interact with amino acid residues in the linker region [38], to be inserted in the hydrophobic region I [39], and to occupy the hydrophobic region II [40,41], respectively (**Fig 1**).

**Fig 2:**
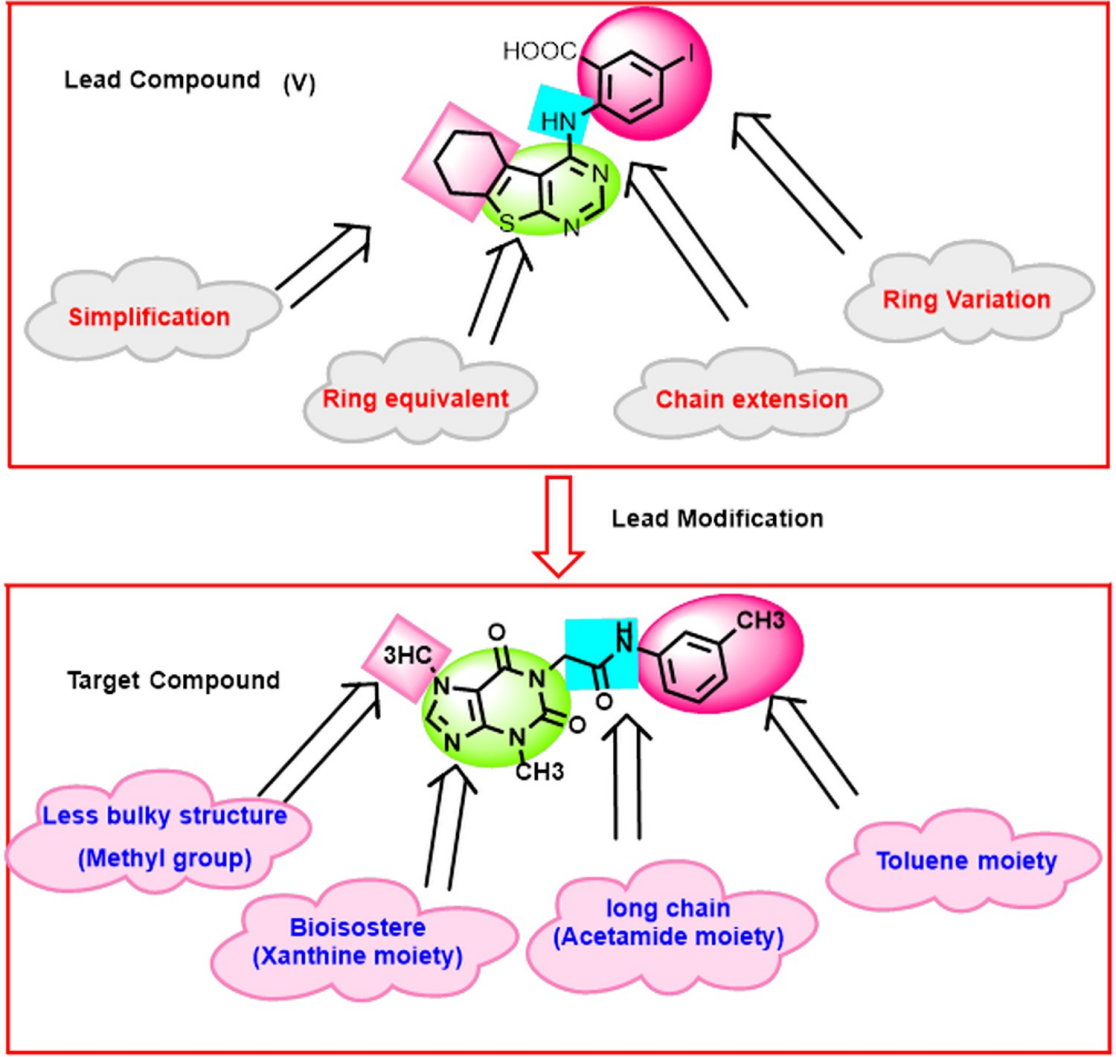
T-1-MTA’s design rationale.

In this work and as an extension of our previous efforts in the discovery of new anti-EGFR agents [36,42–44], compound **V** was used as a lead compound to reach a more promising anticancer agent targeting EGFR. Several chemical modifications were carried out at four positions. The first position is the planar heterocyclic system. We applied the ring variation strategy as the thieno[2,3-*d*] pyrimidine moiety was replaced by a xanthine derivative (3-methyl-3,7-dihydro-1*H*-purine-2,6-dione). The six hydrogen bond (HB) acceptors may facilitate the HB interaction in the adenine binding pocket. Chain extension strategy was applied in the liker region through the replacement of the NH-linker with acetamide moiety. The terminal hydrophobic head (3-iodobenzoic acid) of the lead compound was replaced by toluene moiety) *via* ring variation strategy. A simplification strategy was applied for the hydrophobic tail (cyclohexene) of the lead compound. It was replaced by methyl group at 7-posision of xanthine moiety (**Fig 2**).

## Results and discussions

### 2.1. Chemistry

**Scheme 1:**
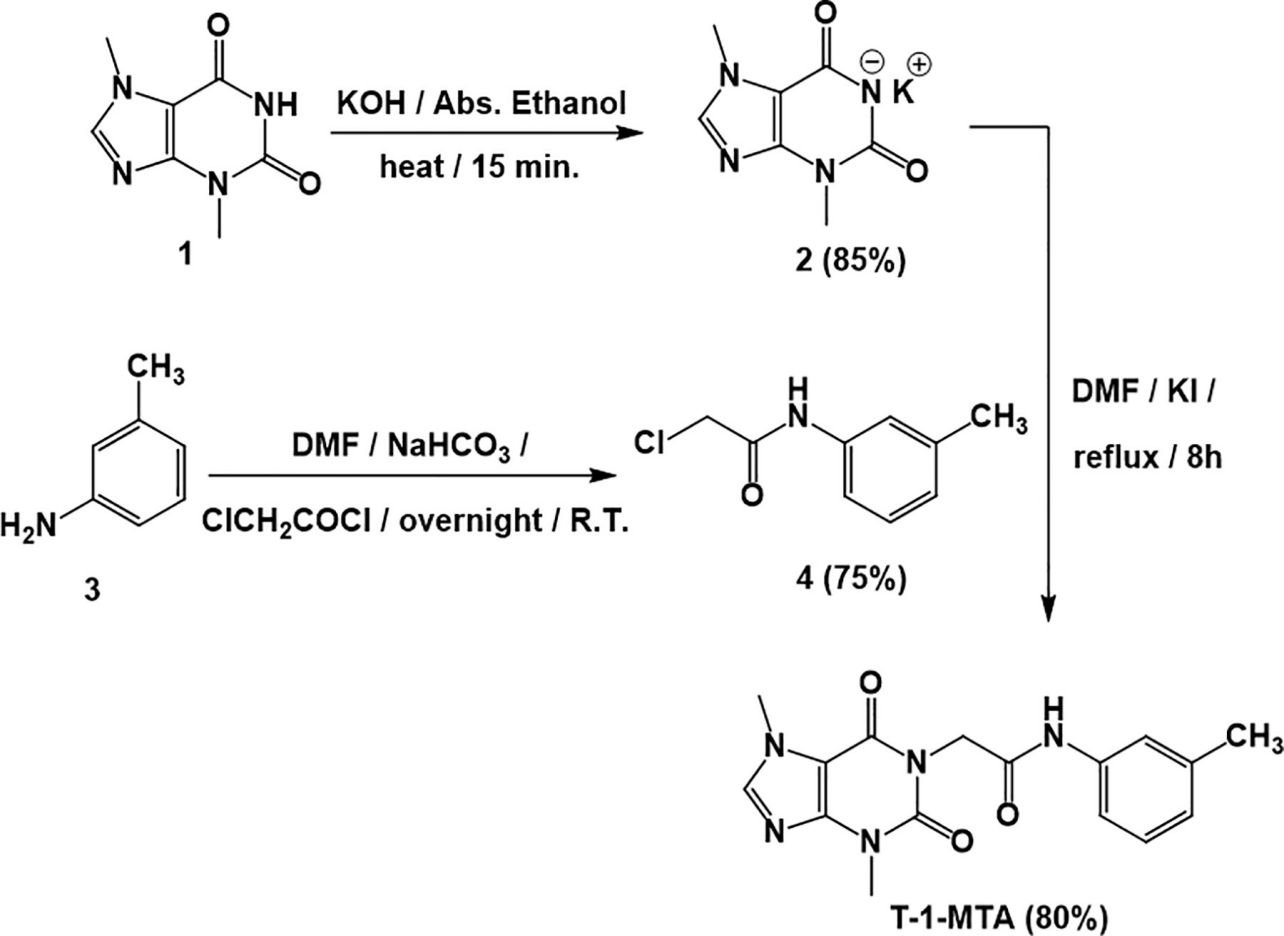
Synthetic pathway of T-1-MTA.

**Scheme 1** depicts the synthetic pathway used in this study to produce target **T-1-MTA**. The potassium salt of 3,7-dimethyl-3,7-dihydro-1*H*-purine-2,6-dione **2** was first obtained by refluxing 3,7-dimethyl-3,7-dihydro-1*H*-purine-2,6-dione (theobromine, **1**) with alcoholic KOH [45,46]. 2-Chloro-*N*-(*m*-tolyl)acetamide **4**, as the key intermediate, was prepared from commercially available *m*-toluidine **3** with chloroacetylchloride in DMF using NaHCO_3_. When equimolar amounts of potassium 3,7-dimethyl-3,7-dihydro-1*H*-purine-2,6-dione **2** and 2-Chloro-*N*-(*m*-tolyl)acetamide **4** were refluxed in DMF containing a sufficient amount of potassium iodide as a catalyst, an expected final product **T-1-MTA** was attained.

The ^1^H NMR spectrum of **T-1-MTA** showed singlet signal at *δ* = 8.07 for CH imidazole and multiplet signals ranging from *δ* 7.41 to 6.87 for aromatic protons besides remarkable singlet signals for the CH_3_ (of *m*-tolyl group) and CH_2_ groups at *δ* = 2.27 and 4.67, respectively. The IR spectrum of the same product revealed absorption bands at 1711, 1662 cm^-1^ corresponding to carbonyl groups and absorption bands at 3255 cm^-1^ corresponding to NH. Regarding the ^13^C NMR spectrum, four shielded signals appeared at 43.84, 33.66, 29.90, and 21.62 ppm corresponding to CH_2_ and the three CH_3_ groups, respectively.

### 2.2. Molecular docking

**Fig 3:**
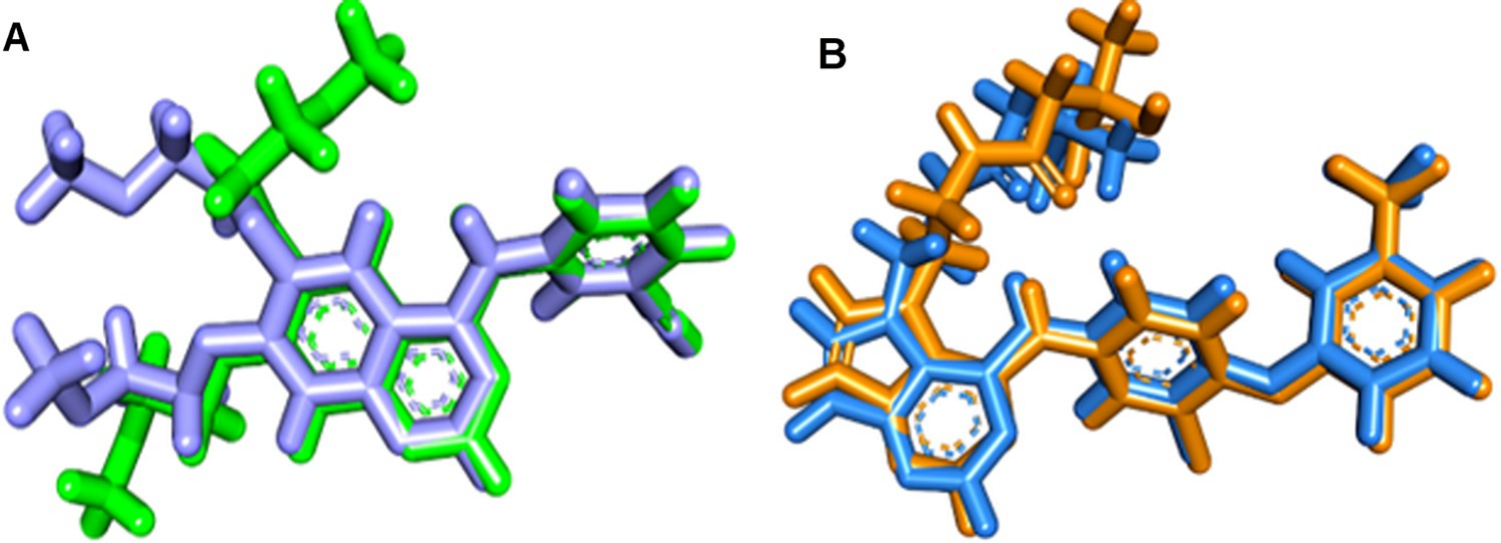
**A:** Validation of wild EGFR using erlotinib as co-crystallized ligand and **B:** Validation of mutant EGFR using TAK-285 as co-crystallized ligand.

The examined proteins’ X-ray structures (EGFR^WT^; PDB: 4HJO and EGFR^T790M^; PDB: 3W2O) were acquired from the Protein Data Bank (PDB, http://www.pdb.org). First, the docking protocol was verified for both wild and mutant EGFR and the RMSD results were 1.20 and 1.15 Å, respectively **Fig 3.**

**Fig 4:**
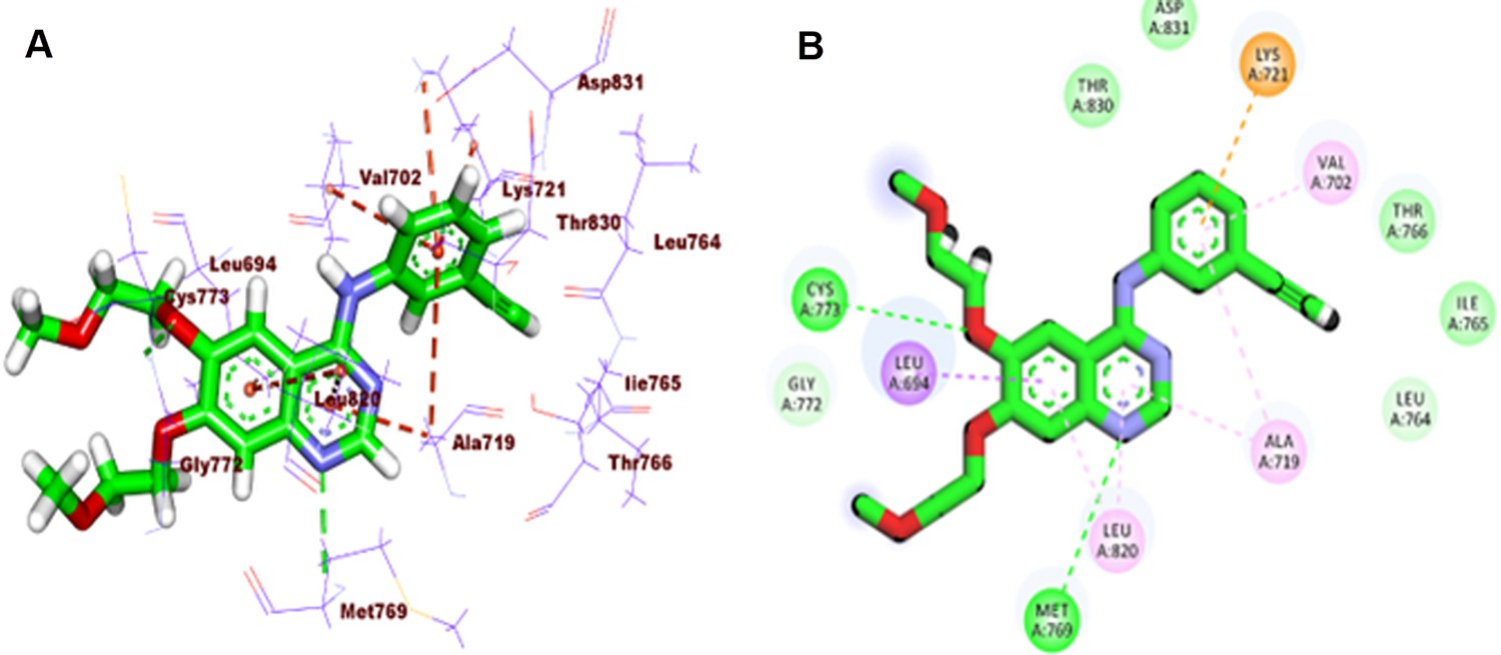
**A:** 3D and **B:** 2D close view of erlotinib EGFR^WT^.

**Fig 5:**
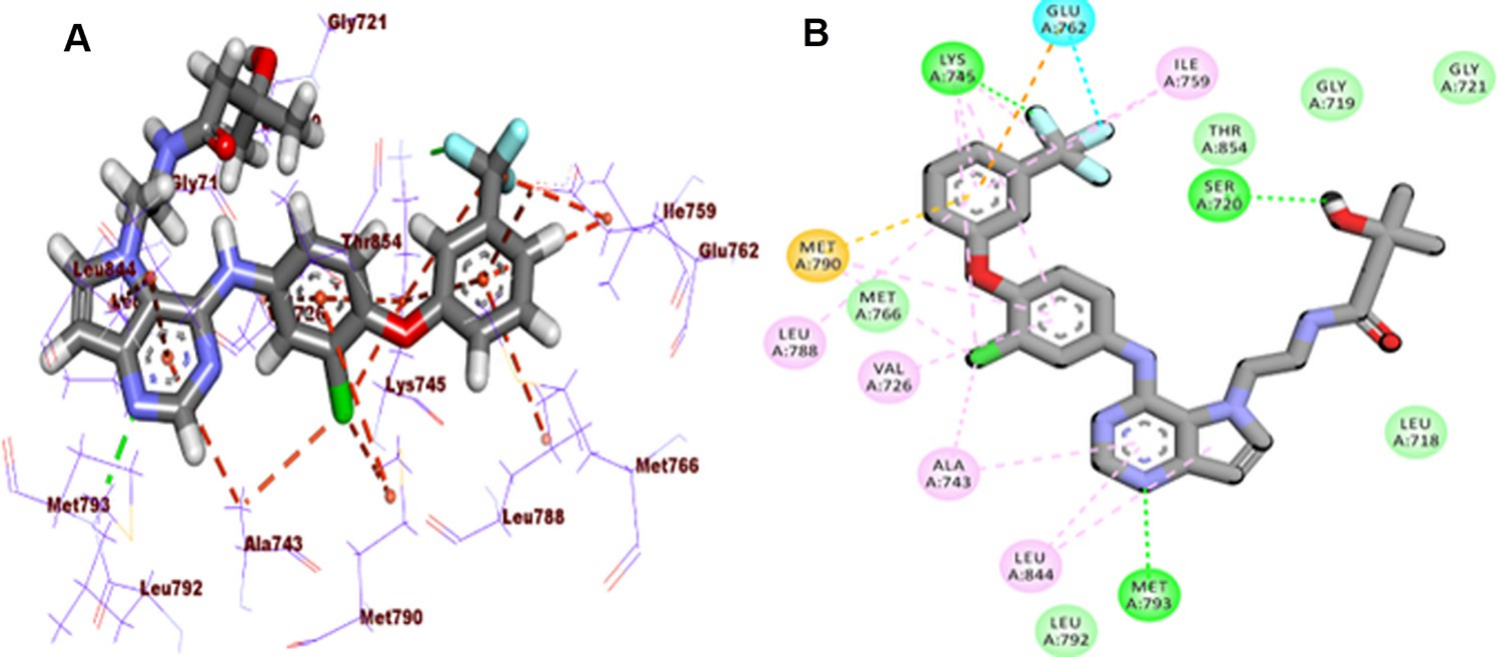
3D and 2D close view of TAK-285 into EGFR^T790M^.

Erlotinib, as a native inhibitor for EGFR^WT^, revealed an affinity value of -20.50 kcal/mol. The binding pattern of erlotinib revealed a key HB with Met769 (2.11 A˚) in addition to four hydrophobic interactions (HI) in the adenine pocket and three HIs with Ala719 and Val702, and Lys721 in the hydrophobic pocket (**Fig 4**). TAK-285, as a native inhibitor for EGFR^T790M^, presented a binding energy of -7.20 kcal/mol. The binding pattern of TAK-285 revealed a key HB with Met793 (2.44 A˚) through the pyrimidine moiety in the adenine pocket. The later moieties (3-(trifluoromethyl)phenoxy and *N*-ethyl-3-hydroxy-3-methylbutanamide moieties) were fixed in the hydrophobic pocket *via* a network of HIs with Lys745, Ile759, Met790, Val726, and Ala743, and Leu844 (**Fig 5**).

**Fig 6:**
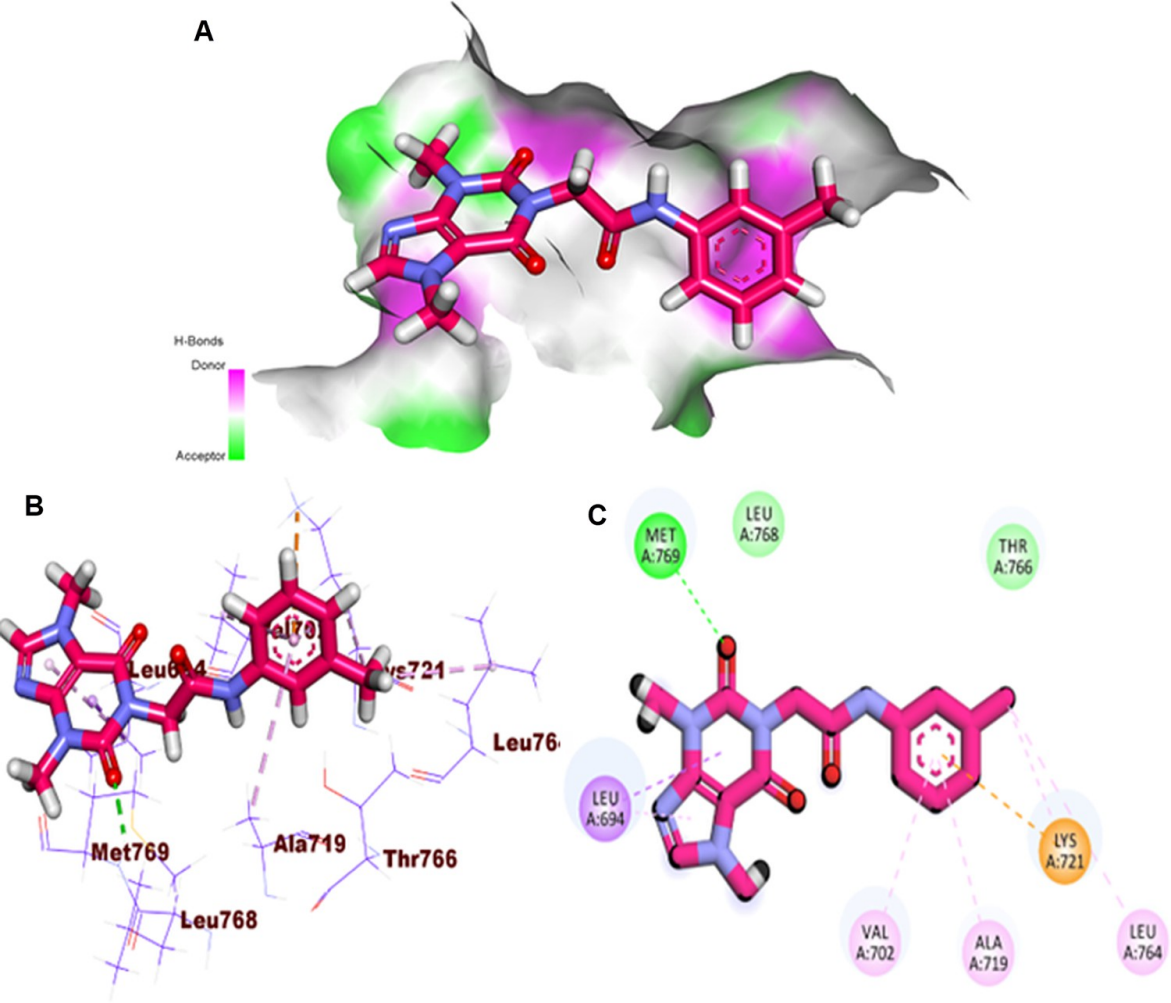
**A:** Mapping Surface (MS), **B:** 3D, and **C:** 2D close view of **T-1-MTA** inside the EGFR^WT^.

Regarding the EGFR^WT^, a comparable affinity value to erlotinib was obtained by **T-1-MTA** (-20.45 kcal/mol). Additionally, it interacts with the EGFR^WT^ active site similar to erlotinib and adopts the same orientation. Besides, the 3,7-dimethyl-2,6-dioxo-2,3,6,7-tetrahydro-1*H*-purine arm formed a crucial HB with Met769 besides two HIs with Lue694 inside the adenine pocket. On the other side, five HIs with Leu764, Ala719, Val702, and Lys721 were achieved *via* the *m*-tolyl moiety in the conserved hydrophobic pocket. The methyl group at 7-posision of xanthine moiety failed to form HIs in the hydrophobic pocket II **(Fig 6).**

**Fig 7:**
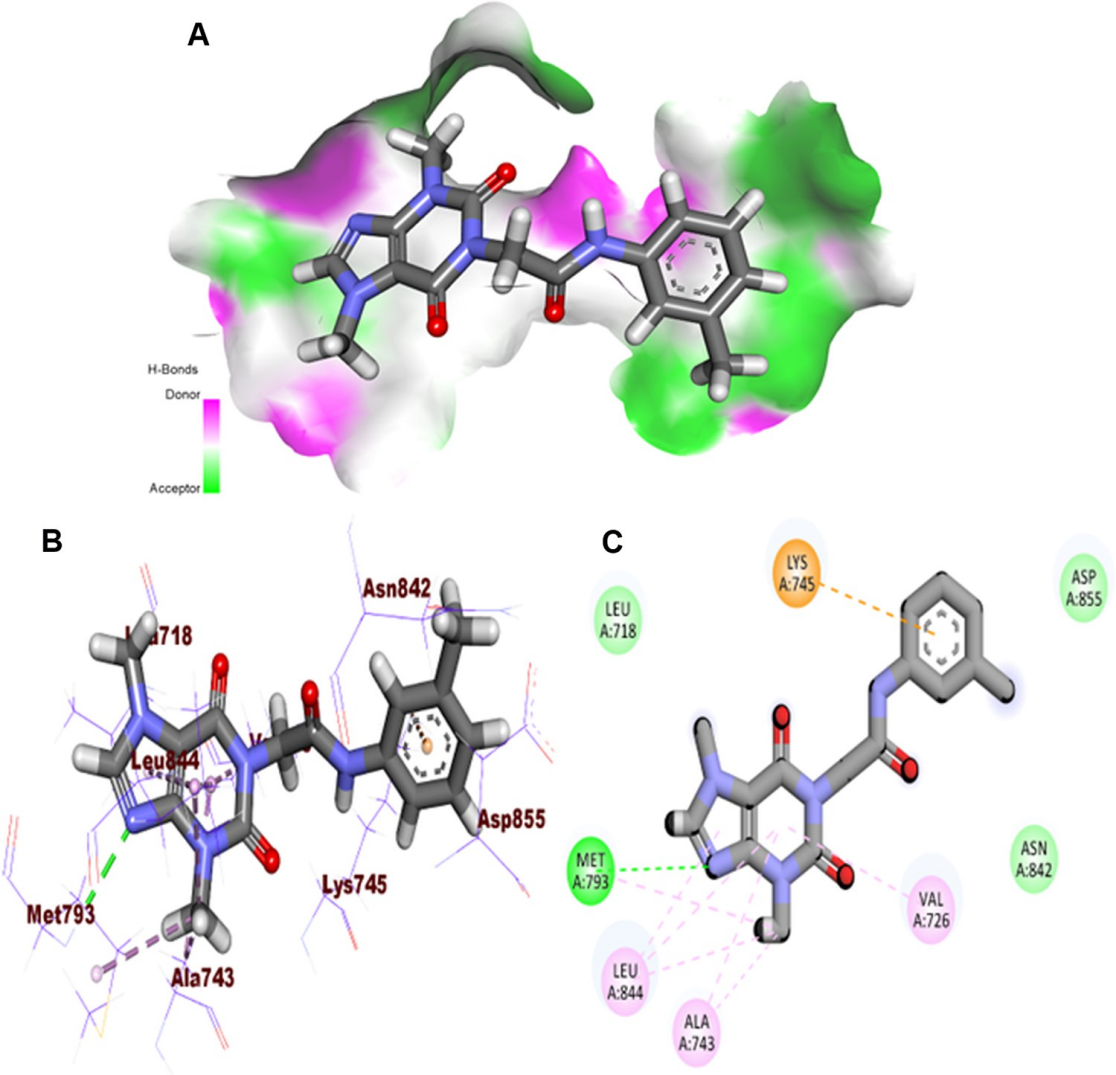
**A:** MS, **B:** 3D, and **C:** 2D close view of **T-1-MTA** with EGFR^T790M^.

Regarding the EGFR^T790M^, **T-1-MTA** (binding energy of -6.95 kcal/mol.) was tacked onto the catalytic site similarly to the positive control, TAK-285. In the adenine pocket, six pi-pi bonds with Leu844, Ala743, and Met79 were accomplished through the 3,7-dimethyl-2,6-dioxo-2,3,6,7-tetrahydro-1*H*-purine arm. Also, in the same region, a crucial HB with Met793 was observed. Additionally, in the hydrophobic pocket, the *m*-tolyl moiety was buried to form one electrostatic interaction with Lys745 **Fig 7.**

### 2.3. MD simulations

**Fig 8:**
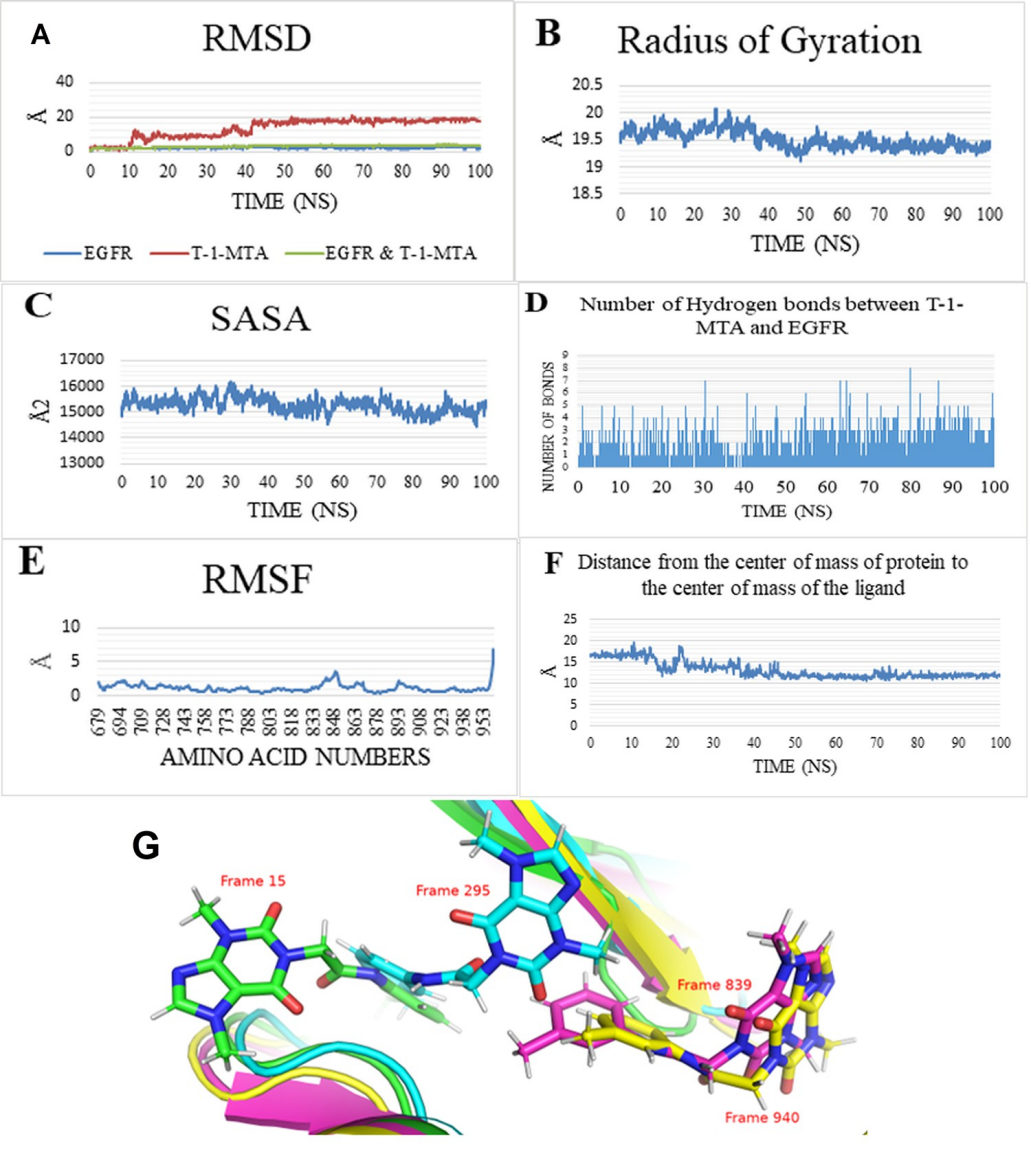
MD measurements calculated for a 100 ns. A) RMSD, B) RoG, C) SASA, D) HBBs’ change between the **T-1-MTA** and EGFR, E) RMSF, F) Center of Mass distance between the compound **T-1-MTA** and EGFR, and G) shows the positions of the compound **T-1-MTA** at different snapshots of the trajectory. **T-1-MTA** is in stick representation while the protein at the same snapshots is in cartoon representation.

The MD analyses obtained on a 100 ns production run showing an overall system stability. The RMSD plot (**Fig 8A**) showed a stable trend for the EGFR only and the EGFR_**T-1-MTA** complex that were represented as blue and green curves showings averages of 2.16 Å and 2.97 Å, respectively. Moreover, the RMSD of the **T-1-MTA** (red) showed three states during the whole trajectory. The first 10 ns show an average of 2.16 Å before spiking to an average of 9.43 Å for the next 30 ns. Moreover, the last 60 ns show a large stable average value of 17.72 Å. The reason for this increase in the RMSD values of the compound **T-1-MTA** is due to the translational movement of the compound **T-1-MTA** relative to the protein as shown in **Fig 8**G which compares between the positions of the ligand at 1.5 ns (green sticks), 29.5 ns (cyan sticks), 83.9 ns (magenta sticks), and 94 ns (yellow sticks). The RoG (**Fig 8B**), SASA and (**Fig 8C**) HB show a stable protein fluctuation with an average of 19.51 Å, and 15285 Å^2^, respectively. The change in HBs between the **T-1-MTA** and EGFR (**Fig 8D**) shows that there is, approximately, at least one HB formed during the first 40 ns and it increases to at least two bonds during the rest of the simulation. The amino acids’ fluctuation was depicted in the RMSF plot (**Fig 8E**) showing low values of fluctuation (less than 2 Å) excepting the free C-terminal and the loop region E842:Y845 reaching 7 Å, and 3.5 Å, respectively. During the simulation time, the distance between the center of mass of compound **T-1-MTA** and the center of mass of EGFR protein shows a similar trend to the RMSD values of the ligand (three states) (**Fig 8F**). It started with an average of 16.72 Å for the first 15 ns before slightly decreasing to an average of 14.02 Å for the next 25 ns (from 15 ns to 40 ns). Finally, the last 60 ns showed an average value of 11.87 Å showing a stable interaction (**Fig 8G**).

### 2.4. MM-GBSA studies

**Fig 9:**
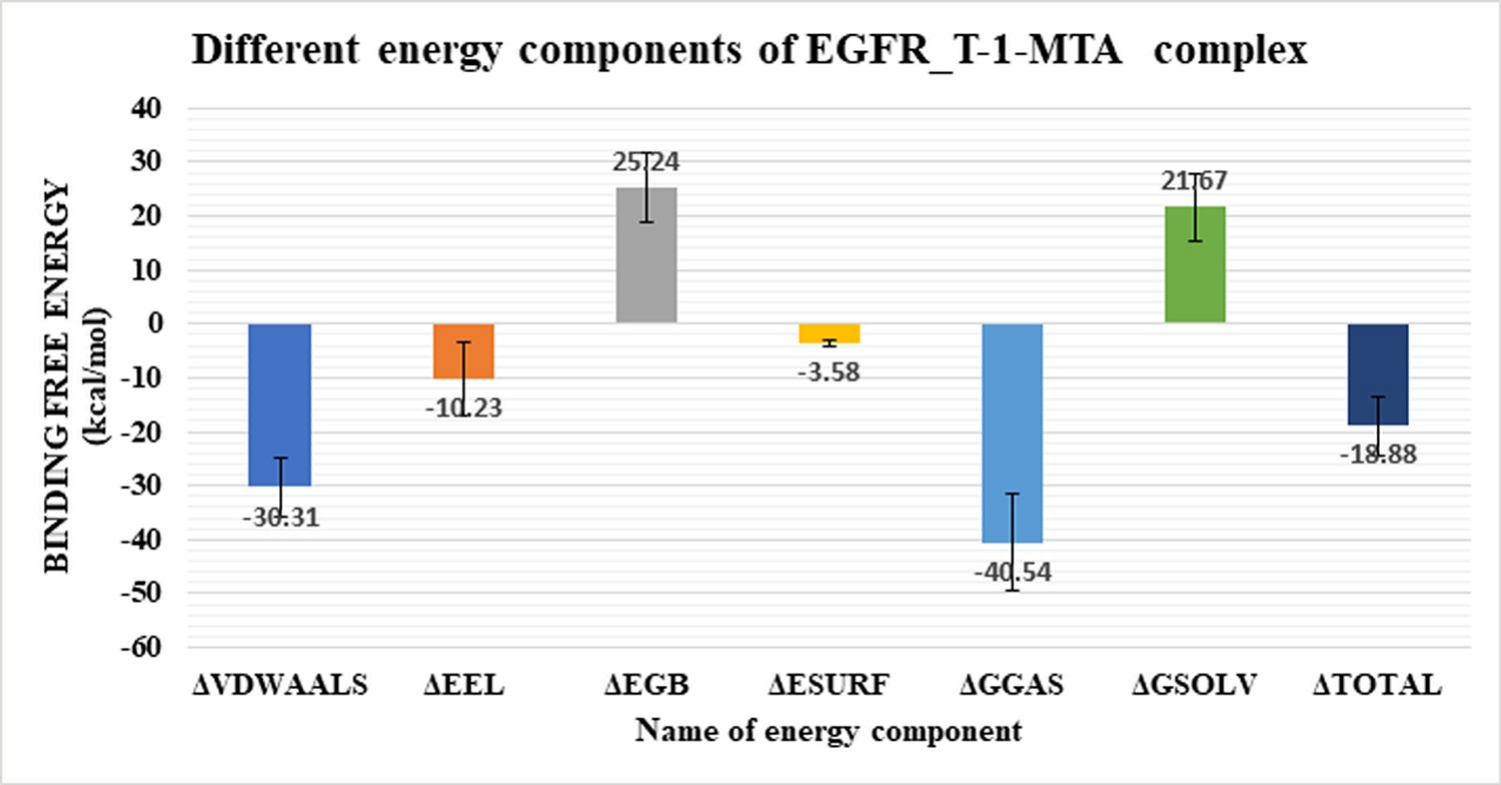
Energetic components of EGFR-T-1-MTA complex. Bars represent the standard deviation.

**Fig 10:**
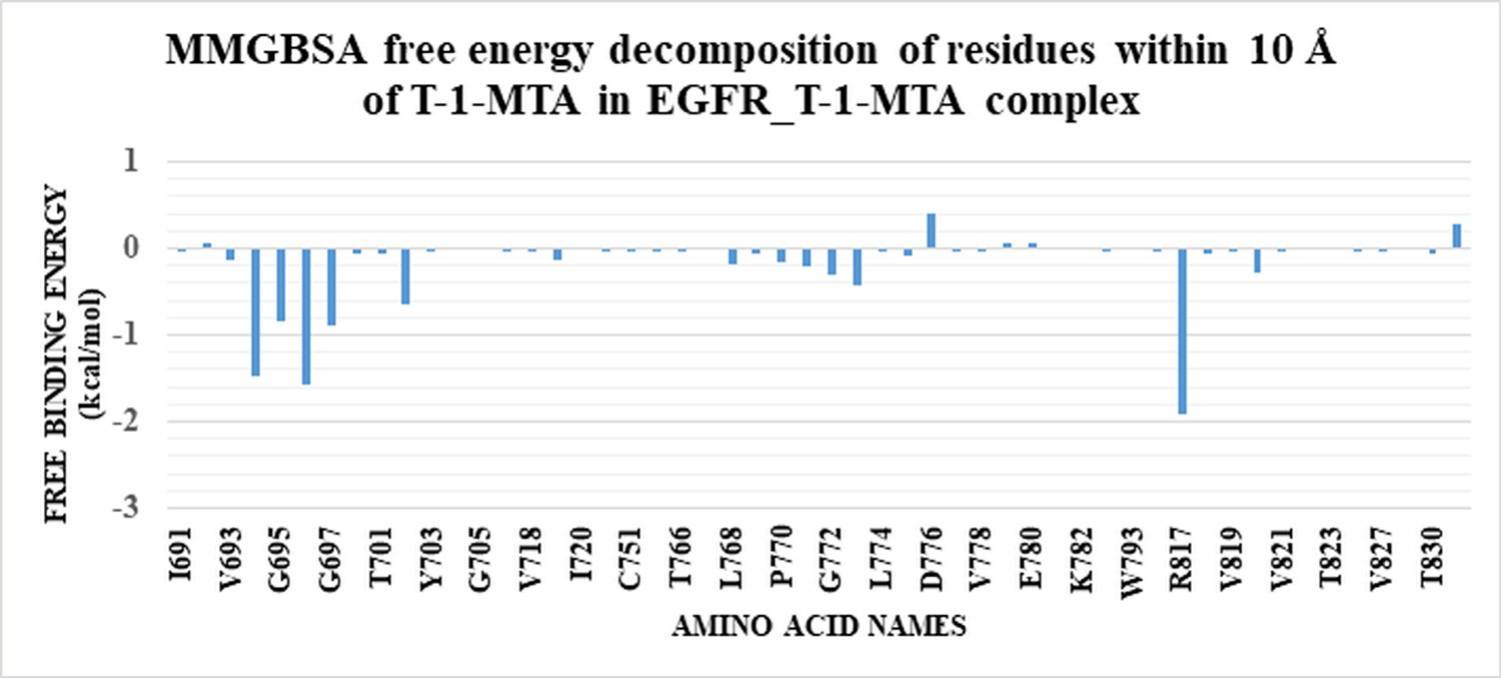
Binding energy decomposition of the EGFR_T-1-MTA complex.

The binding free energy of the EGFR_**T-1-MTA** complex was further analyzed deeply by the MM-GBSA analysis. As **Fig 9** shows, the EGFR_**T-1-MTA** complex had a total binding energy of an average value of -18.88 kcal/Mol. The various forms of energy that contribute to binding of the EGFR_**T-1-MTA** complex were analyzed to be. Van Der Waals interaction, electrostatic interaction with average values of -30.31 kcal/Mol and-10.23 kcal/Mol, respictively. Moreover, we performed an energy- decomposition analysis as shown in **Fig 10** to identify the amino acids that had the highest contribution to the binding (1 nm or better). L694 (-1.48 kcal/Mol), S696 (-1.56 kcal/Mol), and R817 (-1.9 kcal/Mol) are the amino acids that exhibited the best contributions (better or less than -1 kcal/Mol).

### 2.5. Protein-Ligand Interaction Profiler (PLIP) studies

**Fig 11:**
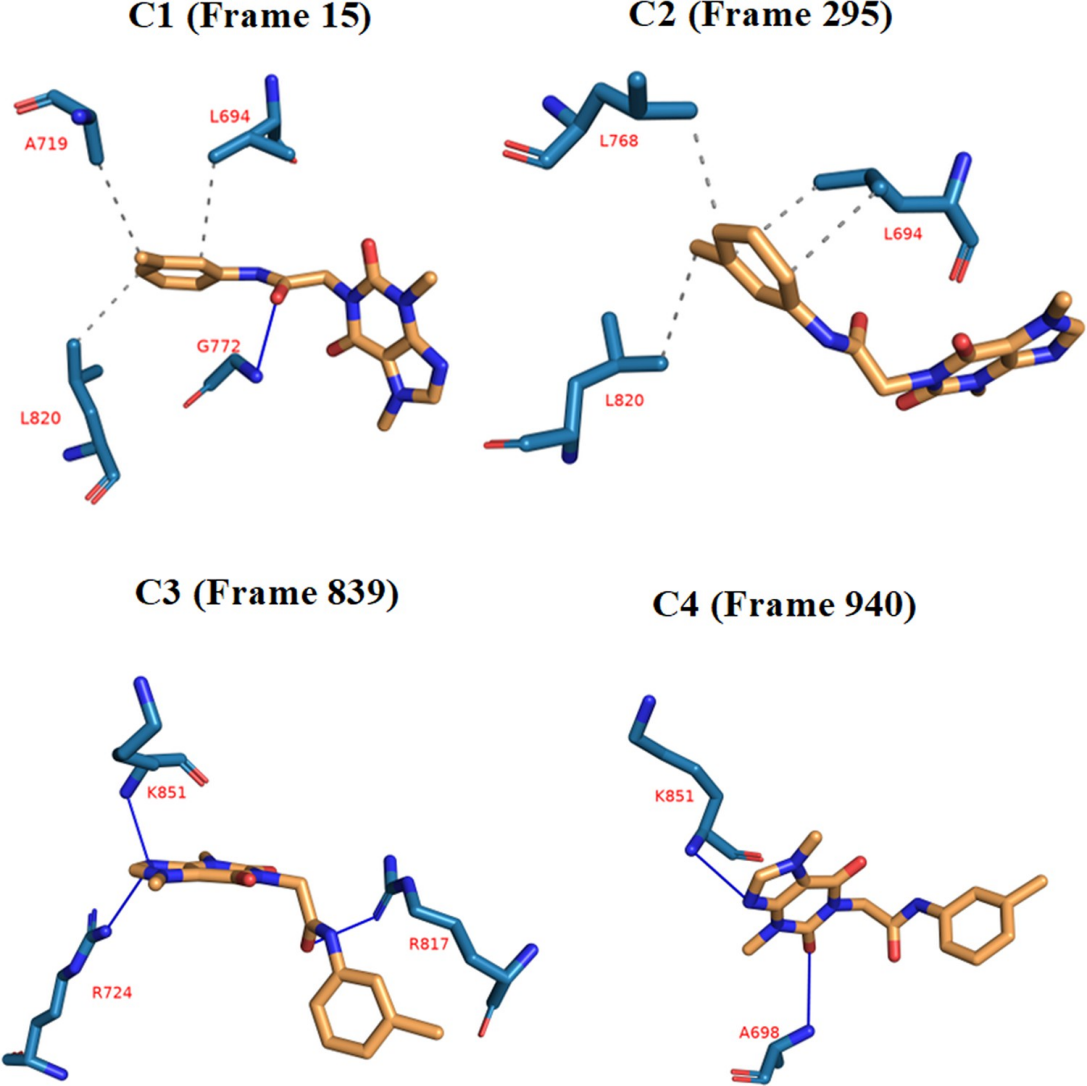
The variation (types and numbers) of interactions of the EGFR_T-1-MTA complex produced from PLIP. HB: Blue solid line, HI: Dashed grey line, green dashed lines: Pi-Stacking interaction, amino acids: Blue sticks, and **T-1-MTA**: Orange sticks.

After that, to obtain a representative frame for each cluster of the EGFR_**T-1-MTA** complex, the obtained trajectory was clustered. The elbow method was used to automatically choose the number of clusters, as described in the methodologies section, and this resulted in four clusters. The PLIP website was used to determine the number and types of interactions between **T-1-MTA** and EGFR for each cluster representative (**Table 1**). As can be seen, HIs have a similar overall number of interactions in all the clusters compared to the HBs (7 HIs vs. 6 HBs). Additionally, a.pse file was generated to understand the 3D conformations of **T-1-MTA** as well as its interaction against the EGFR (**Fig 11**).

**Table 1:** Shows the number and types of interactions detected from the PLIP webserver.

CLUSTER NUMBER	HBS	AMINO ACIDS	HIS	AMINO ACIDS
**C1 (FRAME 15)**	1	G772	3	L694—A719—L820
**C2 (FRAME 295)**	0	None	4	L694 (2)—L768—L820
**C3 (FRAME 839)**	3	R724—R817—K851	0	None
**C4 (FRAME 940)**	2	A698—K851	0	None

### 2.6. DFT studies

In an attempt to clarify the inhibitory activity of **T-1-MTA**, theoretical DFT studies have been explored. The conceptual DFT has been used for understanding the electronic structure of the prepared molecule to determine its structural features which has far-reaching consequences on the molecules’ reactivity. Hence, the DFT-based reactivity descriptors (global), frontier molecular orbital analysis (FMO), and surface potential maps have been investigated to explore the reactivity of the prepared compound.

### Geometry optimization

**Fig 12:**
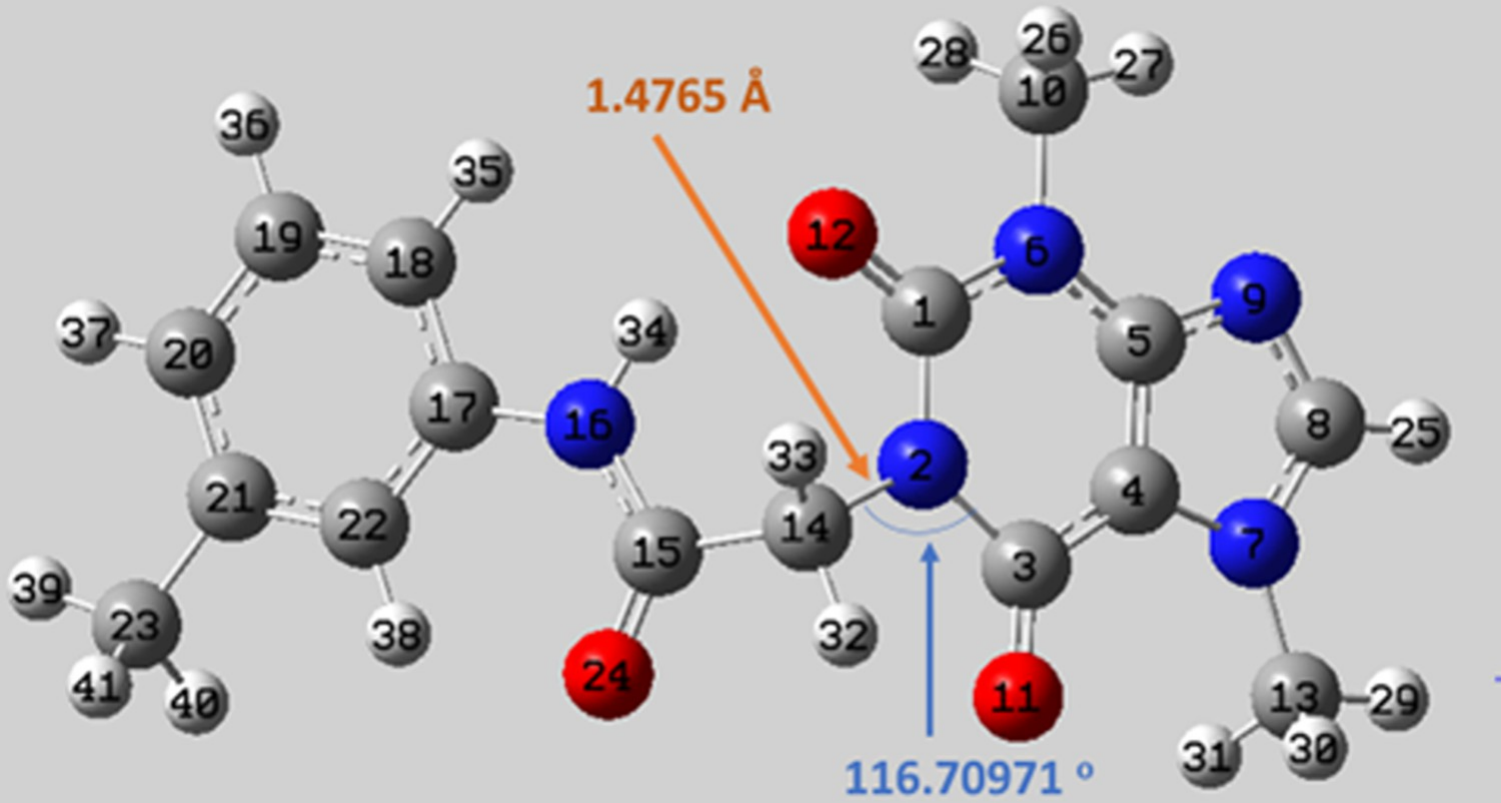
The optimized chemical structure of T-1-MTA.

The reactivity of **T-1-MTA** is mainly determined by its chemical structure, so the structure is fully optimized and computed using DFT. The single bond length **N2-C14** is 1.4765 Å, whereas the **C14-N2-C3** bond angle is 116.70971° as given in **Fig 12** at the B3LYB/6-311G++(d,p) level. The computed ground total energy (TE) is -30470.0 eV whereas the dipole moment (Dm) value is 5.9956 Debye which indicated a strong ability of interaction within the chemical system.

### Frontier molecular orbital analysis (FMO) analysis

**Fig 13:**
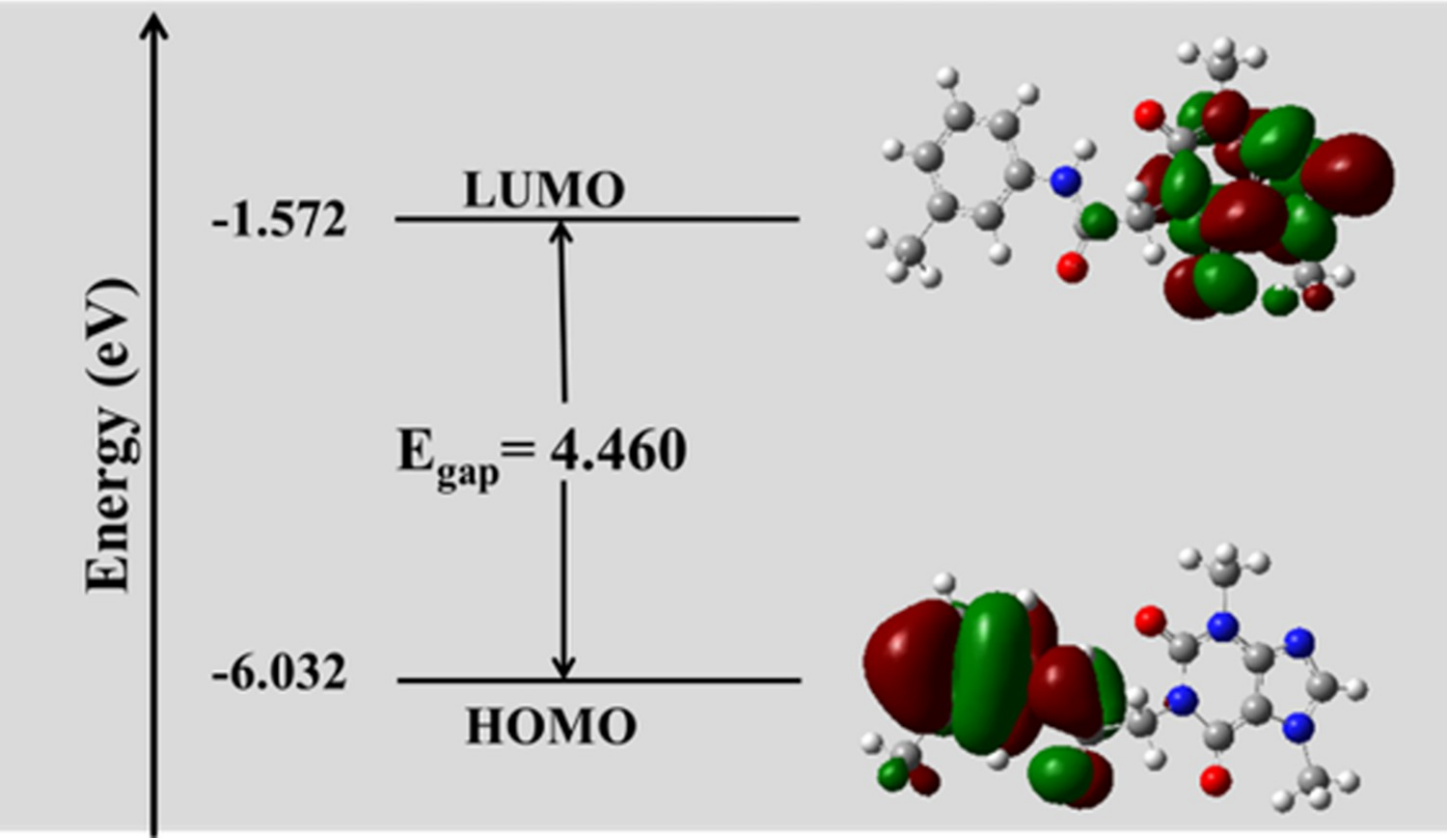
FMO analysis of T-1-MTA.

Border molecular orbitals in a molecule play a vital role in the electric properties as the system with a smaller value of energy gap between the border orbitals (E_gap_ = E_LUMO_-E_HOMO_) should be more reactive than one having a greater E_gap_. Fortunately, **T-1-MTA** reported a smaller E_gap_ value, so the electronic movement between the border orbitals; LUMO and HOMO, could occur easily [47]. The nodal properties of HOMO-LUMO orbitals of the studied heterocyclic molecule in Fig **13** are presented and show the strong orbital overlap, delocalization, and the low number of nodal planes. Hard molecules have a high HOMO-LUMO gap, and soft molecules have a smaller HOMO-LUMO gap. The value of E_gap_ is given in **Fig 13** and indicated that **T-1-MTA** is considered soft and the electronic transition (HOMO-LUMO) within the molecule is easy [48]. The quantum chemical parameters such as ionization potential (IP) and electron affinity (EA) were calculated and listed in **Table 2**.

**Table 2:** The calculated global reactivity indices and energetic parameters for T-1-MTA.

IP	EA	μ (eV)	χ (eV)	η (eV)	σ (eV)	ω (eV)	Dm (Debye)	TE (eV)	ΔN_max_	ΔE (eV)
-6.032	-1.572	-3.802	3.802	2.230	0.448	16.122	5.996	-30470.0	1.705	-16.122

#### Global reactive indices and total density of state (TDOS)

Based on the density functional theory (DFT) concept, global reactivity parameters are essential tools for comprehending the behavior of any chemical molecular structure. Such global reactivity indices depend on the value of E_gap_. In **Table 2**, the static global properties of **T-1-MTA**, namely the electrophilicity (ω), maximal charge acceptance (N_max_), energy change (ΔE), chemical potential (μ), global chemical softness (σ), global electronegativity (χ), global chemical hardness (η), and electron affinity (EA) of **T-1-MTA** are presented after calculating using Koopmans’ theory. The results in **Table 2** indicated that **T-1-MTA** is treated as soft within the nucleophilicity and electrophilicity scales [49].

**Fig 14:**
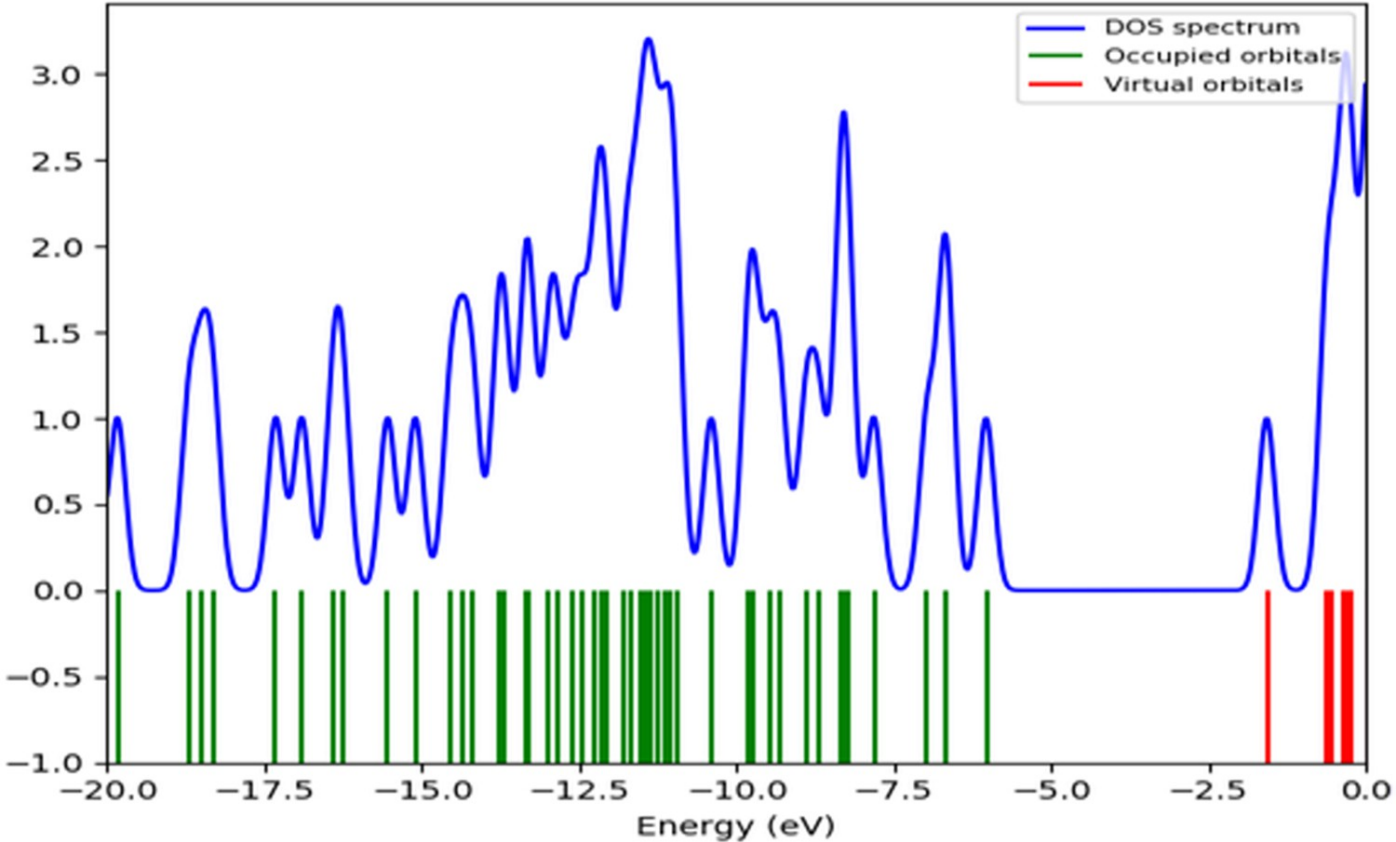
The TDOS spectrum of T-1-MTA.

The density of states and the distribution function probability determined by the occupied states per unit volume are important to provide an accurate description best than frontier molecular orbitals. The TDOS spectrum of **T-1-MTA** in **Fig 14** depicted that the highest electronic intensity is located in the occupied orbitals under the HOMO orbital. Also, the TDOS spectrum confirmed the narrow HOMO-LUMO gap.

#### 2.6.4. Molecular surface potential maps

**Fig 15:**
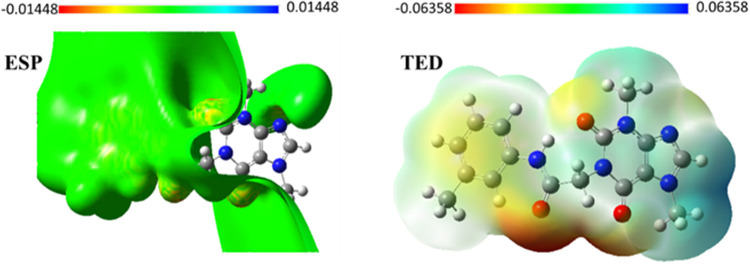
TED and ESP maps of T-1-MTA.

Molecular electrostatic surface potential discovers the relationship between the electronic distribution over the molecule surface and its binding ability. The molecular electrostatic potential explains and predicts the noncovalent interactions. Also, it finds the positive and negative domains of the electrostatic potential with low and high electron densities, respectively. The quantitative electrostatic surface potential (ESP) and total electrostatic density (TED) maps of **T-1-MTA** are demonstrated in **Fig 15** after analysis of the optimized ground-state geometry. It appears that there are red regions indicating the negative potential is localized over the electronegative atoms such as O. The positive potential domains (blue color) are localized on the hydrogen atoms of purine moiety. The areas with moderate electron density values are shown with yellow color and localized on the phenyl ring. It can be predicted that the positive region on the purine ring of **T-1-MTA** will interact strongly with the negative region of the target and the negative areas at oxygen atoms will form strong interactions with areas of positive potential at the target. Also, it can be predicted that there is a strong attraction between the most positive region of **T-1-MTA** and the negative region of the target. The most negative region located around the oxygen atoms can also form a strong interaction with the positive region of the target. This implies that the difference in the distribution of electronic charges could result in enhancing the inhibition reactivity of **T-1-MTA** towards EGFR.

### 2.7. ADMET profiling study

**Fig 16:**
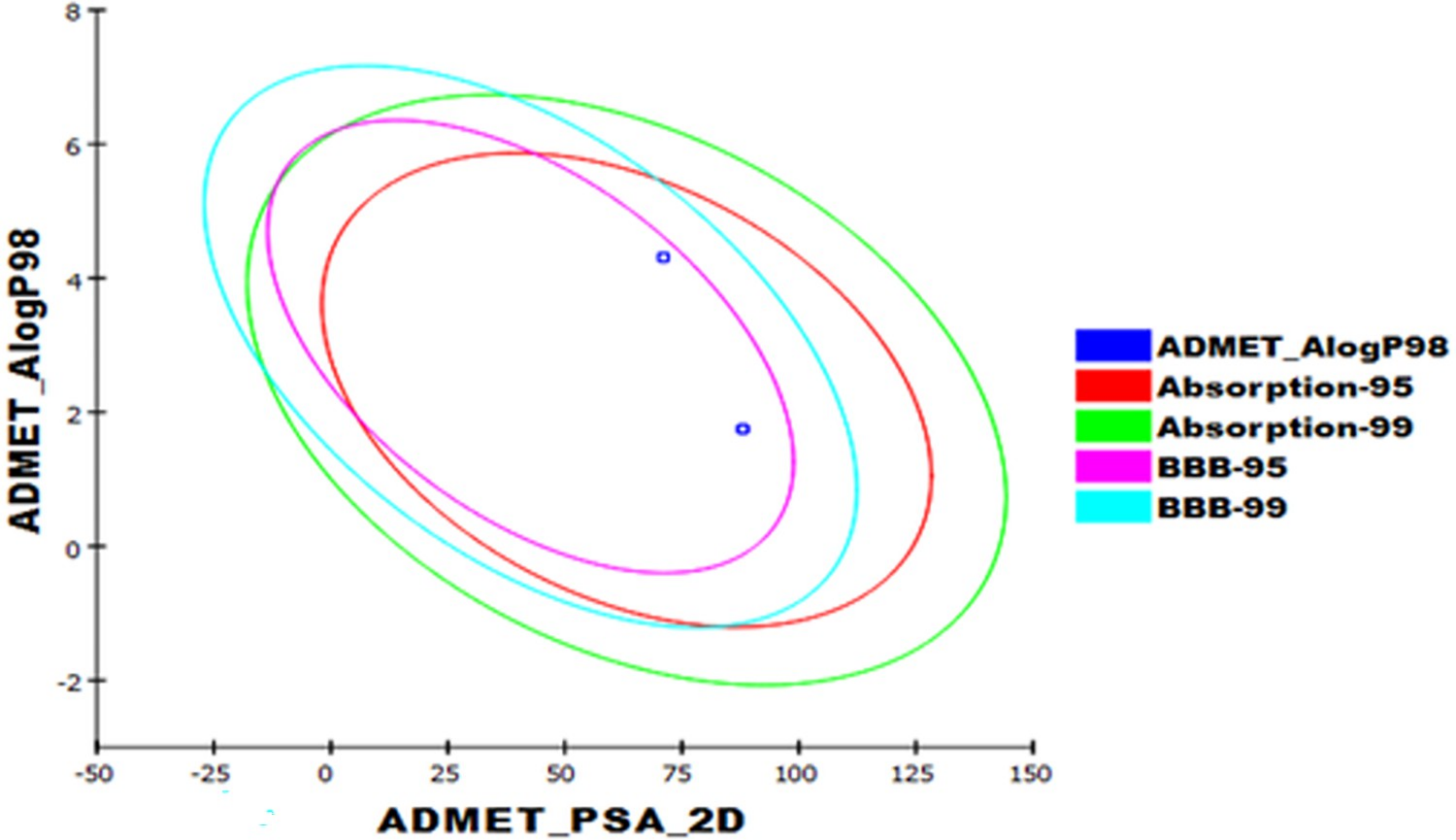
Computational prediction of ADMET parameters for T-1-MTA and erlotinib.

The approval of any new compound as a marketed drug is based on a pharmacokinetic evaluation in addition to its biological activity. So, analyzing the ADME properties of a compound at the early stages should keep the discovery process from being delayed [50]. Although ADMET studies *in vitro* can investigate the properties of the absorbent, distribution, metabolism, excretion, and toxicity of drugs, *in silico* studies are advantageous because of their ability of saving cost, time, effort in addition to the regulations restricting the use of animals [51]. Computing ADMET parameters using Discovery is used to determine the ADMET parameters for **T-1-MTA** against erlotinib. Interestingly, the obtained results of **T-1-MTA** comparing erlotinib (**Fig 16** and **Table 3**) showed a high likeness degree as it was anticipated to have a low potential to pass the BBB. Additionally, hepatotoxicity (HT) and the inhibition of cytochrome P-450 (CYP2D6-I) were expected to be absent. Also, **T-1-MTA** levels of aqueos solubility (AS) and intestinal absorption (IA) were computed as good.

**Table 3:** ADMET parameters for T-1-MTA and erlotinib.

Comp.	BBB	AS	IA	HT	CYP2D6-I	Plasma protein binding
**T-1-MTA**	Low	Good	Good	Non-toxic	Non-inhibitor	< 90%
erlotinib	High	Low		toxic		>90%

### 2.8. *In silico* toxicity studies

For a drug to be developed successfully, toxicity assessment at the early stages must be done in order to control the possibility of failure in the clinical stage [52]. The *in silico* approach to toxicity assessment is promising being accurate and avoiding ethical and resource constraints in the in vitro and *in vivo* phases of toxicity development [53]. *In silico* prediction of toxicity basically uses the structure-activity relationship (SAR)-predicting toxicity. In detail, the computer compares the chemical properties of the examined molecules against the structural properties of tens of thousands of compounds of reported safety or toxicity [54]. Employing the Discovery studio software, eight toxicity models were used to estimate **T-1-MTA**’s toxicity in comparison to erlotinib. Providentially, **T-1-MTA** expressed very good and safe values in the carried-out models (**Table 4**)

**Table 4:** *In silico* toxicity studies of T-1-MTA and erlotinib.

Comp.	FDA Rodent Carcinogenicity (Rat- female)	Carcinogenic Potency TD_50_ (Mouse) ^1^	Ames Mutagenicity	Rat Maximum Tolerated Dose (Feed) ^2^	Rat Oral LD_50_ ^2^	Rat Chronic LOAEL^2^	Skin Irritancy	Ocular Irritancy
T-1-MTA	Non-Carcinogen	111.107	Non-Mutagen	0.018	4.712	0.020	Mild	Mild
erlotinib		39.771	Non-Mutagen	0.083	0.662	0.036	Non-Irritant	Mild

^1^ Unit: mg/kg /day.

^2^ Unit: g/kg.

### 2.9. Biological evaluation

#### 2.9.1. *In vitro* EGFR inhibition

**Fig 17:**
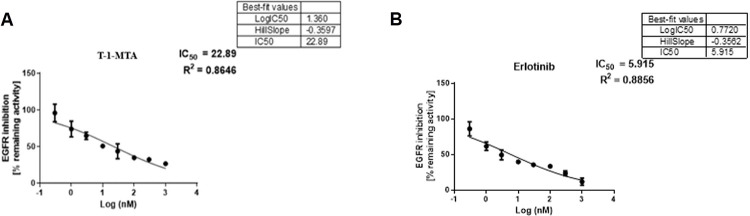
*In vitro* EGFR- inhibition potentialities of T-1-MTA (A) and erlotinib (B).

For the purpose of examining the design and the computational outcomes that clearly demonstrated **T-1-MTA**’s significant affinity for EGFR, **T-1-MTA**’s inhibitory ability was assessed *in vitro* against the EGFR protein (**Fig 17**). The obtained inhibition value (22.89 nM) was near to erlotinib’s value, and the resulting *in vitro* results confirmed **T-1-MTA** ’s suppressive potential.

### 2.9.2 Cytotoxicity and safety

*In vitro* cytotoxicity assessment was performed for **T-1-MTA** using compared to erlotinib as demonstrated in **Table 5**. The obtained IC_50_ values of **T-1-MTA** against A549 and HCT-116 malignant cells were 22.49 and 24.97 μM, respectively. **T-1-MTA**’s anticancer potential was close to that of erlotinib.

**Table 5:** *In vitro* anti-proliferative activities of T-1-MTA.

Comp.	*In vitro* cytotoxicity IC_50_ (μM) ^a^			A549 (SI)	HCT-116 (SI)	EGFR IC_50_ (nM)
	A549	HCT-116	WI-38			
**T-1-MTA**	22.49	24.97	55.14	2.4	2.2	22.89
**Erlotinib**	6.73	16.35	31.17	4.6	1.9	5.91

^**a**^ Data are presented as the mean of the IC_50_ values of triplicate experiments.

**Fig 18:**
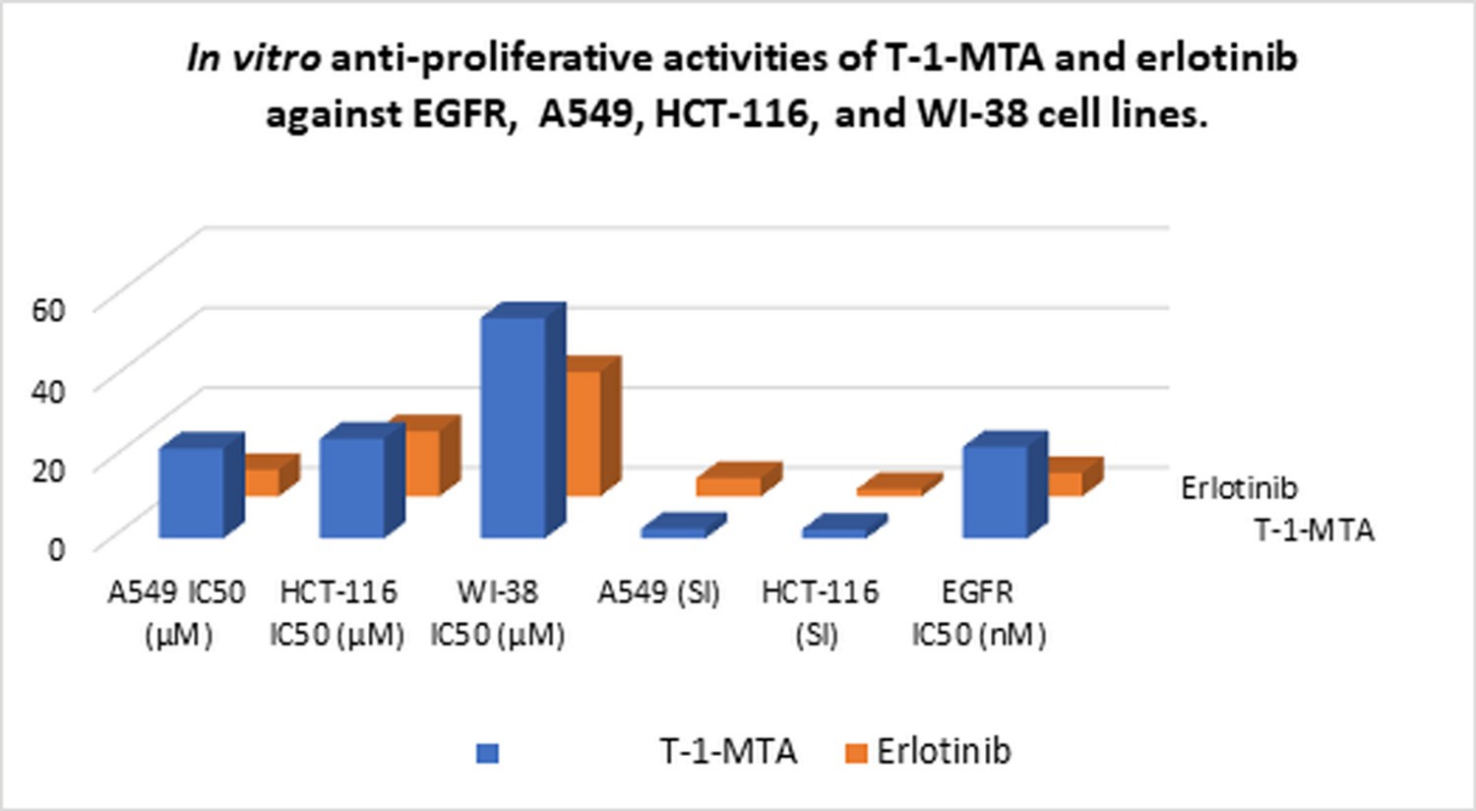
*In vitro* anti-proliferative and safety assessments of T-1-MTA and erlotinib.

As a confirmation of the computed safety pattern of **T-1-MTA** and to explore its selectivity, **T-1-MTA** was tested against the W138 human normal cell line. **T-1-MTA** showed a high IC_50_ value of 55.14 μM as well as very high selectivity indexes (SI) of 2.4 and 2.2 against the two cancer cell lines, respectively (**Fig 18**).

#### 2.9.3. Cell cycle analysis and apoptosis assay

**Fig 19:**
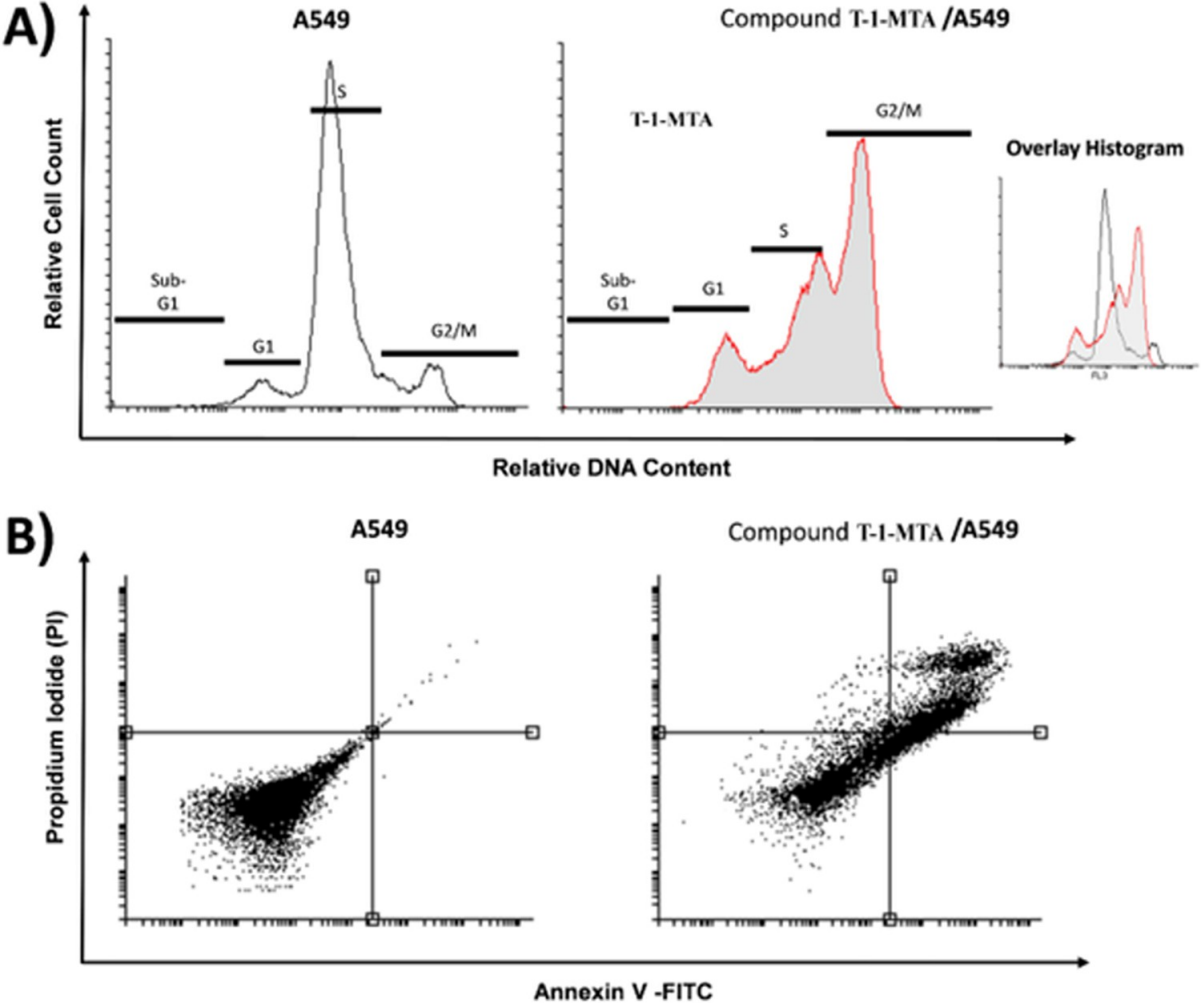
Flow cytometric analysis of cell cycle phases and apoptosis. (**A**) The representative histograms show the cell cycle distribution of control (A549), and cells treated with 22.49 μM (IC_50_ value) of **T-1-MTA** for 72h. (**B**) Flow cytometric charts of apoptosis in A549 cells exposed to **T-1-MTA** (22.49 μM) for 72 h.

Firstly, the cell cycle phases of A549 after **T-1-MTA’**s treatment was analyzed by flow cytometry according to the reported method before [55,56]. A concentration of 22.49 μM of **T-1-MTA** was added to A549 cells for 72 h. Then, the cancer’s cell cycle was investigated. Interestingly, **T-1-MTA** decreased the percentage of A549 cells in the Sub-G1 and S phases from 0.75% and 68.17% to 0.36% and 28.60%, respectively. Contraversly, in the G2/M phase, the A549 percent was significantly increased from 18.69 to 49.20 after **T-1-MTA**’s treatment (**Table 6** and **Fig 19**).

**Table 6:** Effect of T-1-MTA on the cell cycle of A549 cells after 72 h treatment.

Sample	Cell cycle distribution (%) ^a^				
	%Sub-G1		%G1	%S	% G2/M
**A549**	0.75 ± 0.27	12.39 ± 5.08		68.17 ± 6.92	18.69 ± 1.56
**T-1-MTA /A549**	0.36 ± 0.17	21.84 ± 1.83		28.60 ± 5.00*	49.20 ± 3.33*

^**a**^ Values are given as mean ± SEM of two independent experiments.

*p < 0.05 indicates statistically significant differences from the corresponding control (A549) group in unpaired *t*-tests.

To verify the apoptotic effects of **T-1-MTA,** the apoptosis percentage in the A549 cells was examined by Annexin V and PI double stains after it was subjected of 22.49 μM of **T-1-MTA** for 72 h [57,58]. Interestingly, **T-1-MTA** reduced the viable cancer cell count. Comparing control, **T-1-MTA** induced higher ratio of apoptotic cells. Also, **T-1-MTA** caused increased the apoptotic cells’ percentage significantly in the early stage of apoptosis (from 0.07% to 21.24%) as well as the late stage of apoptosis (from 0.73% to 37.97%). Also, the necrosis percentage was elevated to be 1.78, compared to 0.04% in the control cells (**Fig 19** & **Table 7**). In conclusion, **T-1-MTA** successfully arrested the A549 cell cycle at the G2/M phase causing cytotoxic potentialities that may be connected to apoptosis.

**Table 7:** Effect of T-1-MTA on stages of the cell death process in A549 cells after 72 h treatment.

Sample	Viable ^a^ (Left Bottom)	Apoptosis ^a^		Necrosis ^a^ (Left Top)
		Early (Right Bottom)	Late (Right Top)	
**A549**	99.16 ± 0.05	0.07 ± 0.01	0.73 ± 0.07	0.04 ± 0.02
**T-1-MTA / A549**	39.01 ± 4.152	21.24 ± 1.07**	37.97 ± 6.02*	1.78 ± 0.45

^**a**^ Values are given as mean ± SEM of two independent experiments.

*p < 0.05, and **p < 0.01 indicate statistically significant difference from the corresponding control (A549) group in unpaired *t*-tests.

## Conclusion

According to the essential structural features of EGFR inhibitors, a new lead theobromine-derived candidate, **T-1-MTA** has been designed. An anti-EGFR potential of the **T-1-MTA** was showed by molecular docking and verified by six MD simulations (over an100 ns), three MM-GBSA, and three DFT studies. Likely, computational ADMET studies indicated a general drug-likeness and safety. The biological evaluation confirmed the *in silico* results as **T-1-MTA** showed EGFR inhibitory activity with IC_50_ value of 22.89 nM. In addition, it exerted cytotoxic properties against A549 and HCT-116 cell lines with IC_50_ values of 22.49 and 24.97 μM, respectively. Moreover, **T-1-MTA** showed high selectivity indices towards the tumor cells. Also, the apoptotic potential of **T-1-MTA** was confirmed by the flow cytometry analysis. The obtained *in silico* and *in vitro* outputs are considered a step in the way to finding a cure through more deep investigations and or chemical modifications.

## Experimental

### 4.1. Chemistry

**4.1.1.** All apparatus used in analyses of T-1-MTA were illustrated in the supplementary section (**S1**) in S1 Data detailed explanations.

**Fig 20:**
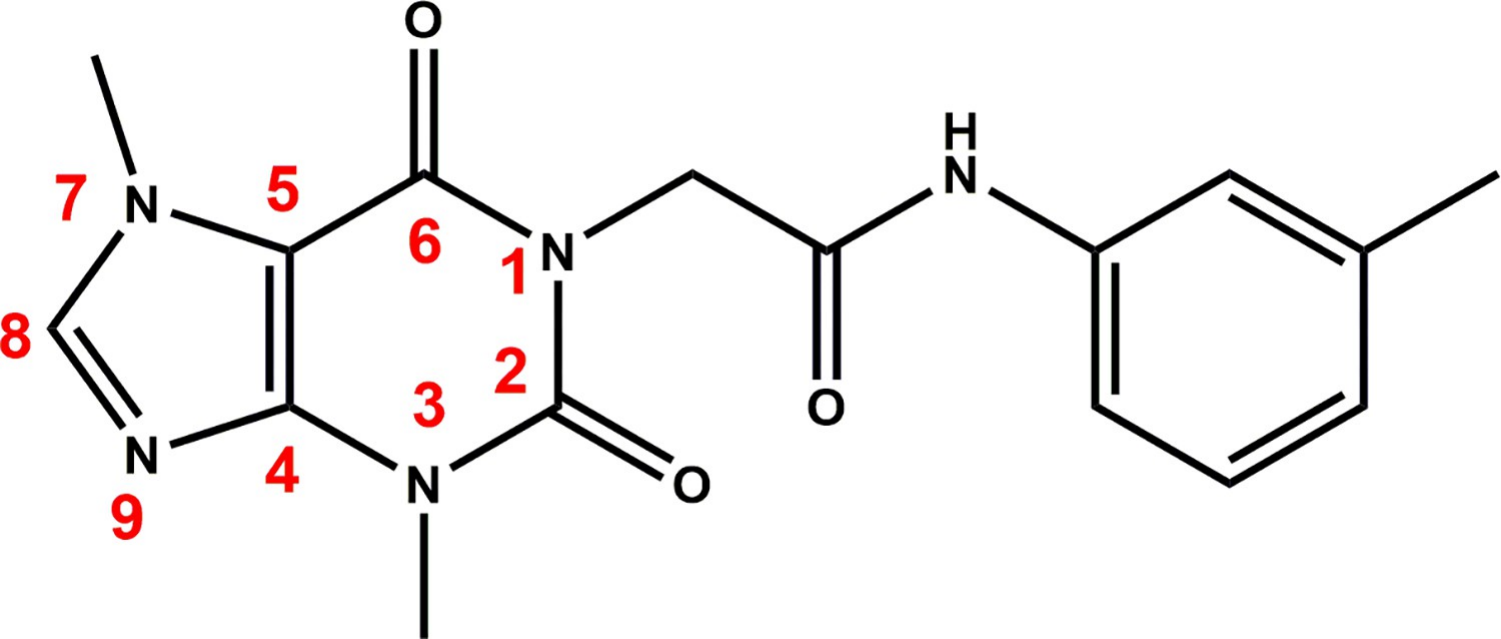
T-1-MTA.

**4.1.2. Synthesis of T-1-MTA.** 2-Chloro-*N*-(*m*-tolyl)acetamide **4** (0.001 mol, 0.21g) was added to a solution of the potassium 3,7-dimethyl-3,7-dihydro-1*H*-purine-2,6-dione **2** (0.001 mol, 0.25g) in DMF (10 mL), and the mixture was heated in a water bath for 8 h. After being poured onto ice water (200 mL), the reaction mixture was gently stirred for certain time. To afford **T-1-MTA (Fig 20)**, the obtained ppt was filtered, water washed, and crystallized from methanol.

Off-white crystal **(**yield, 80%); m. p. = 233–235°C; IR (KBr) *ν* cm^-1^: 3255, 3143 (NH), 3073 (CH aromatic), 2965, 2923 (CH aliphatic), 1711, 1662 (C = O); ^1^H NMR: δ 10.20 (s, 1H, NH), 8.07 (s, 1H, CH imidazole), 7.41 (s, 1H, Ar-H), 7.35 (d, *J* = 8.1 Hz, 1H, Ar-H), 7.19 (t, *J* = 7.8 Hz, 1H, Ar-H), 6.87 (d, *J* = 7.5 Hz, 1H, Ar-H), 4.67 (s, 2H, CH_2_), 3.89 (s, 3H, CH_3_ at position 7 of purine), 3.44 (s, 3H, at position 3 of purine), 2.27 (s, 3H, CH_3_ of methyl phenyl); ^13^C NMR: δ 166.10, 154.64, 151.36, 148.93, 143.67, 139.15, 138.45, 129.08, 124.50, 120.06, 116.67, 107.03, 43.84, 33.66, 29.90, 21.62. For C_16_H_17_N_5_O_3_ (327.34).

### 4.2. Docking studies

Was operated for **T-1-MTA** by MOE2014 software. The supplementary section (**S2**) in S1 Data includes a detailed explanation.

### 4.3. MD simulations

Was operated for **T-1-MTA** by the CHARMM-GUI web server and GROMACS 2021 [24,59]. The supplementary section (**S3**) in S1 Data includes a detailed explanation.

### 4.4. MM-GBSA

Was operated for **T-1-MTA** by the Gmx_MMPBSA package [60]. The supplementary section (**S4**) in S1 Data includes a detailed explanation.

### 4.5. DFT

Was operated for **T-1-MTA** by Gaussian 09 and GaussSum3.0 programs. The supplementary section (**S5**) in S1 Data includes a detailed explanation.

### 4.6. ADMET studies

Was operated for **T-1-MTA** by Discovery Studio 4.0. The supplementary section (**S6**) in S1 Data includes a detailed explanation.

### 4.7. Toxicity studies

Was operated for **T-1-MTA** by Discovery Studio 4.0. The supplementary section (**S7**) in S1 Data includes a detailed explanation.

### 4.8 *In vitro* EGFR inhibition

Was operated for **T-1-MTA** by Human EGFR ELISA kit. The supplementary materials (**S8**) in S1 Data show a comprehensive explanation.

### 4.9. *In vitro* antiproliferative activity

Was operated for **T-1-MTA** by MTT procedure. The supplementary materials (**S9**) in S1 Data show a comprehensive explanation.

### 4.10. Safety assay

Was operated for **T-1-MTA** by MTT procedure utilizing W138 cell lines. The supplementary section (**S10**) in S1 Data includes a detailed explanation.

### 4.11. Cell cycle analysis and apoptosis

Was operated for **T-1-MTA** flowcytometry analysis technique. The supplementary section (**S11 and S12**) in S1 Data includes a detailed explanation.

## Supporting information

S1 Data (PDF)

## References

[1] Abd El-Mageed M.M., Eissa A.A., Farag A.E.-S., Osman E.E.A., Design and synthesis of novel furan, furo [2, 3-d] pyrimidine and furo [3, 2-e][1, 2, 4] triazolo [1, 5-c] pyrimidine derivatives as potential VEGFR-2 inhibitors, Bioorganic Chemistry (2021) 105336. doi: 10.1016/j.bioorg.2021.105336 34530235

[2] Chaudhari P., Bari S., Surana S., Shirkhedkar A., Wakode S., Shelar S., et al S. Ghodke, Logical synthetic strategies and structure-activity relationship of indolin-2-one hybrids as small molecule anticancer agents: An Overview, Journal of Molecular Structure (2021) 131280.

[3] El-Dash Y., Elzayat E., Abdou A.M., Hassan R.A., Novel thienopyrimidine-aminothiazole hybrids: design, synthesis, antimicrobial screening, anticancer activity, effects on cell cycle profile, caspase-3 mediated apoptosis and VEGFR-2 inhibition, Bioorganic Chemistry 114 (2021) 105137. doi: 10.1016/j.bioorg.2021.105137 34237644

[4] Nicholson R.I., Gee J.M.W., Harper M.E., EGFR and cancer prognosis, European journal of cancer 37 (2001) 9–15. 11597399 10.1016/s0959-8049(01)00231-3

[5] Spano J.-P., Lagorce C., Atlan D., Milano G., Domont J., Benamouzig R., et al. Morere, Impact of EGFR expression on colorectal cancer patient prognosis and survival, Annals of oncology 16(1) (2005) 102–108. 15598946 10.1093/annonc/mdi006

[6] Normanno N., De Luca A., Bianco C., Strizzi L., Mancino M., Maiello M.R., et al. Salomon, Epidermal growth factor receptor (EGFR) signaling in cancer, Gene 366(1) (2006) 2–16. 16377102 10.1016/j.gene.2005.10.018

[7] Ayati A., Moghimi S., Salarinejad S., Safavi M., Pouramiri B., A.J.B. c. Foroumadi, A review on progression of epidermal growth factor receptor (EGFR) inhibitors as an efficient approach in cancer targeted therapy, 99 (2020) 103811. 10.1016/j.bioorg.2020.103811 32278207

[8] Eldehna W.M., El Hassab M.A., Elsayed Z.M., Al-Warhi T., Elkady H., Abo-Ashour M.F., et al, Design, synthesis, in vitro biological assessment and molecular modeling insights for novel 3-(naphthalen-1-yl)-4, 5-dihydropyrazoles as anticancer agents with potential EGFR inhibitory activity, Scientific reports 12(1) (2022) 1–13. 35896557 10.1038/s41598-022-15050-8 PMC9329325

[9] Metwaly A.M., Ghoneim M.M., Eissa I.H., Elsehemy I.A., Mostafa A.E., Hegazy M.M., et al, Traditional ancient Egyptian medicine: a review, Saudi Journal of Biological Sciences (2021) 5823–5832. doi: 10.1016/j.sjbs.2021.06.044 34588897 PMC8459052

[10] Han X., Yang Y., Metwaly A.M., Xue Y., Shi Y., Dou D., The Chinese herbal formulae (Yitangkang) exerts an antidiabetic effect through the regulation of substance metabolism and energy metabolism in type 2 diabetic rats, Journal of ethnopharmacology 239 (2019) 111942. doi: 10.1016/j.jep.2019.111942 31075380

[11] Newman D.J., Cragg G.M., Natural products as sources of new drugs from 1981 to 2014, Journal of Natural Products 79(3) (2016) 629–661. doi: 10.1021/acs.jnatprod.5b01055 26852623

[12] Barcz E., Sommer E., Janik P., Marianowski L., Skopinska-Rozewska E., Adenosine receptor antagonism causes inhibition of angiogenic activity of human ovarian cancer cells, Oncology reports 7(6) (2000) 1285–1376. doi: 10.3892/or.7.6.1285 11032931

[13] Kakuyamanee Iwazaki A., Sadzuka Y., Effect of methylxanthine derivatives on doxorubicin transport and antitumor activity, Current drug metabolism 2(4) (2001) 379–395. doi: 10.2174/1389200013338270 11766989

[14] Sultani H.N., Ghazal R.A., Hayallah A.M., Abdulrahman L.K., Abu‐Hammour K., AbuHammad S., et al M.O., Inhibitory effects of new mercapto xanthine derivatives in human mcf7 and k562 cancer cell lines, Journal of Heterocyclic Chemistry 54(1) (2017) 450–456.

[15] Woskresensky A., Ueber das Theobromin, Justus Liebigs Annalen der Chemie 41(1) (1842) 125–127.

[16] A.J.J.L.A.d.C. Woskresensky, Ueber das Theobromin, 41(1) (1842) 125–127.

[17] Fredholm B.B., Smit H.J., Theobromine and the pharmacology of cocoa, Methylxanthines (2011) 201–234. 10.1007/978-3-642-13443-2_7 20859797

[18] Sugimoto N., Miwa S., Hitomi Y., Nakamura H., Tsuchiya H., Yachie A., Theobromine, the primary methylxanthine found in Theobroma cacao, prevents malignant glioblastoma proliferation by negatively regulating phosphodiesterase-4, extracellular signal-regulated kinase, Akt/mammalian target of rapamycin kinase, and nuclear factor-kappa B, Nutrition and cancer 66(3) (2014) 419–423. doi: 10.1080/01635581.2013.877497 24547961

[19] Gil M., Skopińska-Rózewska E., Radomska D., Demkow U., Skurzak H., Rochowska M., et al, Effect of purinergic receptor antagonists suramin and theobromine on tumor-induced angiogenesis in BALB/c mice, Folia Biologica 39(2) (1993) 63–68. 7504997

[20] Barcz E., Sommer E., Sokolnicka I., Gawrychowski K., Roszkowska-Purska K., Janik P., et al, The influence of theobromine on angiogenic activity and proangiogenic cytokines production of human ovarian cancer cells, Oncology reports 5(2) (1998) 517–537. doi: 10.3892/or.5.2.517 9468592

[21] da Rosa R., Schenkel E.P., Bernardes L.S.C., Semisynthetic and newly designed derivatives based on natural chemical scaffolds: moving beyond natural products to fight Trypanosoma cruzi, Phytochemistry Reviews 19(1) (2020) 105–122.

[22] Belal A., Abdel Gawad N.M., Mehany A.B., Abourehab M.A., Elkady H., Al‐Karmalawy A.A., Ismael A.S., Design, synthesis and molecular docking of new fused 1 H-pyrroles, pyrrolo [3, 2-d] pyrimidines and pyrrolo [3, 2-e][1, 4] diazepine derivatives as potent EGFR/CDK2 inhibitors, Journal of Enzyme Inhibition and Medicinal Chemistry 37(1) (2022) 1884–1902. doi: 10.1080/14756366.2022.2096019 35801486 PMC9272933

[23] Taghour M.S., Elkady H., Eldehna W.M., El-Deeb N.M., Kenawy A.M., Elkaeed E.B., et al, Design and synthesis of thiazolidine-2, 4-diones hybrids with 1, 2-dihydroquinolones and 2-oxindoles as potential VEGFR-2 inhibitors: in-vitro anticancer evaluation and in-silico studies, Journal of Enzyme Inhibition and Medicinal Chemistry 37(1) (2022) 1903–1917. doi: 10.1080/14756366.2022.2085693 35801403 PMC9272924

[24] Elkaeed E.B., Youssef F.S., Eissa I.H., Elkady H., Alsfouk A.A., Ashour M.L., et al, Multi-Step In Silico Discovery of Natural Drugs against COVID-19 Targeting Main Protease, International Journal of Molecular Sciences 23(13) (2022) 6912. doi: 10.3390/ijms23136912 35805916 PMC9266348

[25] Belal A., Elanany M.A., Santali E.Y., Al-Karmalawy A.A., Aboelez M.O., Amin A.H., et al, Screening a Panel of Topical Ophthalmic Medications against MMP-2 and MMP-9 to Investigate Their Potential in Keratoconus Management, Molecules 27(11) (2022) 3584. doi: 10.3390/molecules27113584 35684529 PMC9182209

[26] Taghour M.S., Mahdy H.A., Gomaa M.H., Aglan A., Eldeib M.G., Elwan A., et al, Benzoxazole derivatives as new VEGFR-2 inhibitors and apoptosis inducers: design, synthesis, in silico studies, and antiproliferative evaluation, Journal of Enzyme Inhibition and Medicinal Chemistry 37(1) (2022) 2063–2077. doi: 10.1080/14756366.2022.2103552 35875937 PMC9327782

[27] Suleimen Y.M., Jose R.A., Suleimen R.N., Ishmuratova M.Y., Toppet S., Dehaen W., et al A.A., Isolation and In Silico SARS-CoV-2 Main Protease Inhibition Potential of Jusan Coumarin, a New Dicoumarin from Artemisia glauca, Molecules 27(7) (2022) 2281. doi: 10.3390/molecules27072281 35408682 PMC9000794

[28] Eissa I.H., Khalifa M.M., Elkaeed E.B., Hafez E.E., Alsfouk A.A., Metwaly A.M., In Silico Exploration of Potential Natural Inhibitors against SARS-Cov-2 nsp10, Molecules 26(20) (2021) 6151. doi: 10.3390/molecules26206151 34684735 PMC8539059

[29] Elkaeed E.B., Elkady H., Belal A., Alsfouk B.A., Ibrahim T.H., Abdelmoaty M., et al, Multi-Phase In Silico Discovery of Potential SARS-CoV-2 RNA-Dependent RNA Polymerase Inhibitors among 3009 Clinical and FDA-Approved Related Drugs, Processes 10(3) (2022) 530.

[30] Suleimen Y.M., Jose R.A., Suleimen R.N., Arenz C., Ishmuratova M., Toppet S., et al. Eissa, Isolation and In Silico Anti-SARS-CoV-2 Papain-Like Protease Potentialities of Two Rare 2-Phenoxychromone Derivatives from Artemisia spp, Molecules 27(4) (2022) 1216. 35209006 10.3390/molecules27041216 PMC8879996

[31] Bonomi P., Erlotinib: a new therapeutic approach for non-small cell lung cancer, Expert opinion on investigational drugs 12(8) (2003) 1395–1401. doi: 10.1517/13543784.12.8.1395 12882624

[32] Nossier E.S., Alasfoury R.A., Hagras M., El-Manawaty M., Sayed S.M., Ibrahim I.M., et al H., Modified pyrido [2, 3-d] pyrimidin-4 (3H)-one derivatives as EGFRWT and EGFRT790M inhibitors: Design, synthesis, and anti-cancer evaluation, Journal of Molecular Structure (2022) 133971. 10.1080/14756366.2022.2062752 PMC9291687 35821615

[33] Jänne P.A., Yang J.C.-H., Kim D.-W., Planchard D., Ohe Y., Ramalingam S.S., et al, AZD9291 in EGFR inhibitor–resistant non–small-cell lung cancer, New England Journal of Medicine 372(18) (2015) 1689–1699. doi: 10.1056/NEJMoa1411817 25923549

[34] Traxler P., Bold G., Frei J., Lang M., Lydon N., Mett H., et al, Use of a pharmacophore model for the design of EGF-R tyrosine kinase inhibitors: 4-(phenylamino) pyrazolo [3, 4-d] pyrimidines, Journal of medicinal chemistry 40(22) (1997) 3601–3616. doi: 10.1021/jm970124v 9357527

[35] Ducray R., Ballard P., Barlaam B.C., Hickinson M.D., Kettle J.G., Ogilvie D.J., et al, Novel 3-alkoxy-1H-pyrazolo [3, 4-d] pyrimidines as EGFR and erbB2 receptor tyrosine kinase inhibitors, Bioorganic & medicinal chemistry letters 18(3) (2008) 959–962. doi: 10.1016/j.bmcl.2007.12.035 18182285

[36] Elmetwally S.A., Saied K.F., Eissa I.H., Elkaeed E.B., Design, synthesis and anticancer evaluation of thieno [2, 3-d] pyrimidine derivatives as dual EGFR/HER2 inhibitors and apoptosis inducers, Bioorganic chemistry 88 (2019) 102944. doi: 10.1016/j.bioorg.2019.102944 31051400

[37] Zhao Z., Wu H., Wang L., Liu Y., Knapp S., Liu Q., et al, Exploration of type II binding mode: A privileged approach for kinase inhibitor focused drug discovery?, ACS chemical biology 9(6) (2014) 1230–1241. doi: 10.1021/cb500129t 24730530 PMC4068218

[38] Furet P., Caravatti G., Lydon N., Priestle J.P., Sowadski J.M., Trinks U., Traxler P., Modelling study of protein kinase inhibitors: binding mode of staurosporine and origin of the selectivity of CGP 52411, Journal of Computer-Aided Molecular Design 9(6) (1995) 465–472. doi: 10.1007/BF00124317 8789188

[39] Mowafy S., Galanis A., Doctor Z.M., Paranal R.M., Lasheen D.S., Farag N.A., et al, Toward discovery of mutant EGFR inhibitors; Design, synthesis and in vitro biological evaluation of potent 4-arylamino-6-ureido and thioureido-quinazoline derivatives, Biorg. Med. Chem. 24(16) (2016) 3501–3512. 10.1016/j.bmc.2016.05.063 27288180

[40] Gandin V., Ferrarese A., Dalla Via M., Marzano C., Chilin A., Marzaro G., Targeting kinases with anilinopyrimidines: discovery of N-phenyl-N’-[4-(pyrimidin-4-ylamino) phenyl] urea derivatives as selective inhibitors of class III receptor tyrosine kinase subfamily, Scientific reports 5 (2015) 16750. doi: 10.1038/srep16750 26568452 PMC4645160

[41] Liu Y., Gray N.S., Rational design of inhibitors that bind to inactive kinase conformations, Nature Chemical Biology 2(7) (2006) 358–364. doi: 10.1038/nchembio799 16783341

[42] Gaber A.A., Bayoumi A.H., El-Morsy A.M., Sherbiny F.F., Mehany A.B., Eissa I.H., Design, synthesis and anticancer evaluation of 1H-pyrazolo [3, 4-d] pyrimidine derivatives as potent EGFRWT and EGFRT790M inhibitors and apoptosis inducers, Bioorganic chemistry 80 (2018) 375–395. doi: 10.1016/j.bioorg.2018.06.017 29986185

[43] Nasser A.A., Eissa I.H., Oun M.R., El-Zahabi M.A., Taghour M.S., Belal A., et al, Discovery of new pyrimidine-5-carbonitrile derivatives as anticancer agents targeting EGFR WT and EGFR T790M, Organic & biomolecular chemistry 18(38) (2020) 7608–7634. doi: 10.1039/d0ob01557a 32959865

[44] Elzahabi H.S., Nossier E.S., Alasfoury R.A., El-Manawaty M., Sayed S.M., Elkaeed E.B., et al, Design, synthesis, and anti-cancer evaluation of new pyrido [2, 3-d] pyrimidin-4 (3H)-one derivatives as potential EGFRWT and EGFRT790M inhibitors and apoptosis inducers, Journal of Enzyme Inhibition and Medicinal Chemistry 37(1) (2022) 1053–1076. doi: 10.1080/14756366.2022.2062752 35821615 PMC9291687

[45] Elkaeed E.B., Yousef R.G., Elkady H., Alsfouk A.A., Husein D.Z., Ibrahim I.M., et al, New anticancer theobromine derivative targeting egfrwt and egfrt790m: Design, semi-synthesis, in silico, and in vitro anticancer studies, Molecules 27(18) (2022) 5859. doi: 10.3390/molecules27185859 36144596 PMC9500845

[46] Elkaeed E.B., Yousef R.G., Elkady H., Alsfouk A.A., Husein D.Z., Ibrahim I.M., et al A New Theobromine-Based EGFRWT and EGFRT790M Inhibitor and Apoptosis Inducer: Design, Semi-Synthesis, Docking, DFT, MD Simulations, and In Vitro Studies, Processes 10(11) (2022) 2290.

[47] Husein D.Z., Hassanien R., Khamis M.J.R.A., Cadmium oxide nanoparticles/graphene composite: synthesis, theoretical insights into reactivity and adsorption study, 11(43) (2021) 27027–27041. doi: 10.1039/d1ra04754j 35480026 PMC9037664

[48] Hanee U., Rahman M., Matin M.M., Synthesis PASS, In Silico ADMET and thermodynamic studies of some galactopyranoside esters, Physical Chemistry Research 9(4) (2021) 591–603.

[49] Wang T., Husein D.Z.J.E.S., Research P., Novel synthesis of multicomponent porous nano-hybrid composite, theoretical investigation using DFT and dye adsorption applications: disposing of waste with waste, (2022) 1–28. 10.1007/s11356-022-20050-2 35460480

[50] Ferreira L.L., Andricopulo A.D., ADMET modeling approaches in drug discovery, Drug discovery today 24(5) (2019) 1157–1165. doi: 10.1016/j.drudis.2019.03.015 30890362

[51] Norinder U., Bergström C.A., Prediction of ADMET properties, ChemMedChem: Chemistry Enabling Drug Discovery 1(9) (2006) 920–937. 10.1002/cmdc.200600155 16952133

[52] Dearden J.C., In silico prediction of drug toxicity, Journal of computer-aided molecular design 17(2) (2003) 119–127. doi: 10.1023/a:1025361621494 13677480

[53] Idakwo G., Luttrell J., Chen M., Hong H., Zhou Z., Gong P., Zhang C., A review on machine learning methods for in silico toxicity prediction, Journal of Environmental Science and Health, Part C 36(4) (2018) 169–191. doi: 10.1080/10590501.2018.1537118 30628866

[54] Kruhlak N., Benz R., Zhou H., Colatsky T., (Q) SAR modeling and safety assessment in regulatory review, Clinical Pharmacology & Therapeutics 91(3) (2012) 529–534. 22258468 10.1038/clpt.2011.300

[55] Elwan A., Abdallah A.E., Mahdy H.A., Dahab M.A., Taghour M.S., Elkaeed E.B., et al. Alsfouk, Modified benzoxazole-based VEGFR-2 inhibitors and apoptosis inducers: Design, synthesis, and anti-proliferative evaluation, Molecules 27(15) (2022) 5047. 35956997 10.3390/molecules27155047 PMC9370530

[56] Nossier E.S., Alasfoury R.A., Hagras M., El-Manawaty M., Sayed S.M., Ibrahim I.M., et al, Modified pyrido[2,3-d]pyrimidin-4(3H)-one derivatives as EGFRWT and EGFRT790M inhibitors: Design, synthesis, and anti-cancer evaluation, Journal of Molecular Structure (2022) 133971. 10.1080/14756366.2022.2062752 PMC9291687 35821615

[57] Alsaif N.A., Taghour M.S., Alanazi M.M., Obaidullah A.J., Al-Mehizia A.A., Alanazi M.M., et al. Elkady, Discovery of new VEGFR-2 inhibitors based on bis ([1, 2, 4] triazolo)[4, 3-a: 3’, 4’-c] quinoxaline derivatives as anticancer agents and apoptosis inducers, Journal of enzyme inhibition and medicinal chemistry 36(1) (2021) 1093–1114. 34056992 10.1080/14756366.2021.1915303 PMC8168755

[58] Alanazi M.M., Eissa I.H., Alsaif N.A., Obaidullah A.J., Alanazi W.A., Alasmari A.F., et al, Design, synthesis, docking, ADMET studies, and anticancer evaluation of new 3-methylquinoxaline derivatives as VEGFR-2 inhibitors and apoptosis inducers, Journal of Enzyme Inhibition and Medicinal Chemistry 36(1) (2021) 1760–1782. doi: 10.1080/14756366.2021.1956488 34340610 PMC8344243

[59] Elkaeed E.B., Eissa I.H., Elkady H., Abdelalim A., Alqaisi A.M., Alsfouk A.A., et al A Multistage In Silico Study of Natural Potential Inhibitors Targeting SARS-CoV-2 Main Protease, International Journal of Molecular Sciences 23(15) (2022) 8407. 35955547 10.3390/ijms23158407 PMC9369012

[60] Elkaeed E.B., Yousef R.G., Elkady H., Gobaara I.M.M., Alsfouk B.A., Husein D.Z., et al, Design, Synthesis, Docking, DFT, MD Simulation Studies of a New Nicotinamide-Based Derivative: In Vitro Anticancer and VEGFR-2 Inhibitory Effects, Molecules 27(14) (2022) 4606. doi: 10.3390/molecules27144606 35889478 PMC9317904

